# Crystal structure of 7′-(4-chloro­phen­yl)-2′′-(4-meth­oxy­phen­yl)-7′,7a’,7′′,8′′-tetra­hydro-1′*H*,3′*H*,5′′*H*-di­spiro­[indoline-3,5′-pyrrolo­[1,2-*c*]thia­zole-6′,6′′-quinoline]-2,5′′-dione and an unknown solvent

**DOI:** 10.1107/S2056989019000112

**Published:** 2019-01-11

**Authors:** R. Vishnupriya, M. Venkateshan, J. Suresh, R. V. Sumesh, R. Ranjith Kumar, P. L. Nilantha Lakshman

**Affiliations:** a Department of Physics, The Madura College, Madurai - 625 011, India; b Department of Physics, The Madura College, Madurai 625 011, India; c Department of Organic Chemistry, School of Chemistry, Madurai Kamaraj University, Madurai 625 021, India; d Department of Food, Science and Technology, University of Ruhuna, Mapalana, Kamburupitiya 81100, Sri Lanka

**Keywords:** crystal structure, spiro-pyrrolidine, indoline, pyrrolo­thia­zole, iso­quinoline, hydrogen bonding, supra­molecular framework, *SQUEEZE*

## Abstract

The asymmetric unit of the title compound, C_34_H_28_ClN_3_O_3_S, contains two independent mol­ecules (*A* and *B*). They differ essentially in the orientation of the 4-meth­oxy­phenyl ring with respect to the pyridine ring of the quinoline moiety; this dihedral angle is 37.01 (18)° in mol­ecule *A* but only 7.06 (17)° in mol­ecule *B*. In both mol­ecules, the cyclo­hexa­none ring of the iso­quinoline unit has a half-chair conformation. In the pyrrolo­thia­zole ring system, the pyrrolo ring in mol­ecule *A* has a twisted conformation on the N—C fused bond and an envelope conformation in mol­ecule *B* with the N atom as the flap. The thia­zole rings of both mol­ecules have twisted conformations on the N—C fused bond. In the crystal, the *A* mol­ecules are linked by pairs of N—H⋯O hydrogen bonds, forming inversion dimers with an *R* ^2^_2_(8) ring motif. These dimers are linked to the *B* mol­ecules by an N—H⋯N hydrogen bond and a series of C—H⋯O hydrogen bonds, forming layers lying parallel to the (101) plane. The layers are linked by C—H⋯π inter­actions and offset π–π inter­actions [inter­centroid distance = 3.427 (1) Å], forming a supra­molecular framework. The contribution to the scattering from a region of highly disordered solvent mol­ecules was removed with the SQUEEZE routine in *PLATON* [Spek (2015 ▸). Acta Cryst. C71, 9–18]. The solvent formula mass and unit-cell characteristics were not taken into account during refinement.

## Abstract 2

The title compound crystallizes with two independent mol­ecules in the asymmetric unit. They differ essentially in the orientation of the 4-meth­oxy­phenyl ring with respect to the pyridine ring of the quinoline moiety.

## Chemical context   

Pyrazolo (Siminoff *et al.*, 1973 ▸; Zheng *et al.*, 2006 ▸) quinoline ring systems are a privileged class of nitro­gen-containing heterocycles endowed with significant biological activities. Quinoline derivatives have been reported to possess many inter­esting pharmacological activities and they are characteristic components of a large number of biologically active compounds. The wide spectrum of biological effects of these kind of compounds includes anti-viral (Billker *et al.*, 1998 ▸; Roma *et al.*, 2000 ▸; Chen *et al.*, 2001 ▸), and anti­fungal (Vargas *et al.*, 2003 ▸; Singh *et al.*, 1996 ▸) agents. In view of their significance, the primary goal of the X-ray diffraction analysis of the title compound was to obtain detailed information on the structural conformation that may be useful in understanding the chemical reactivity of such compounds.
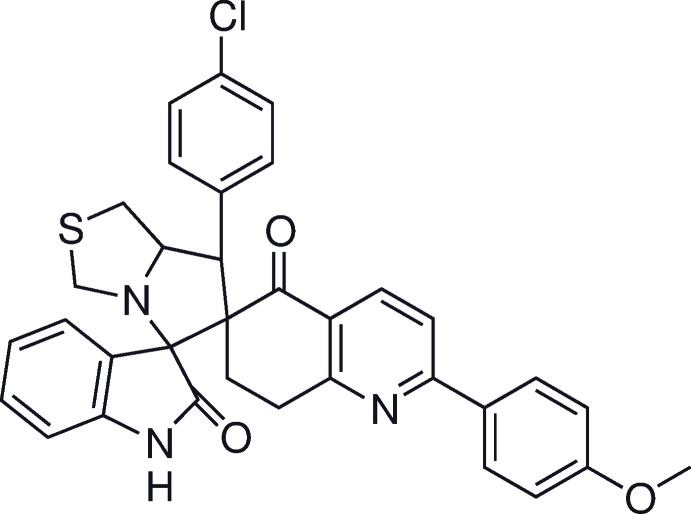


## Structural commentary   

**Figure 1:**
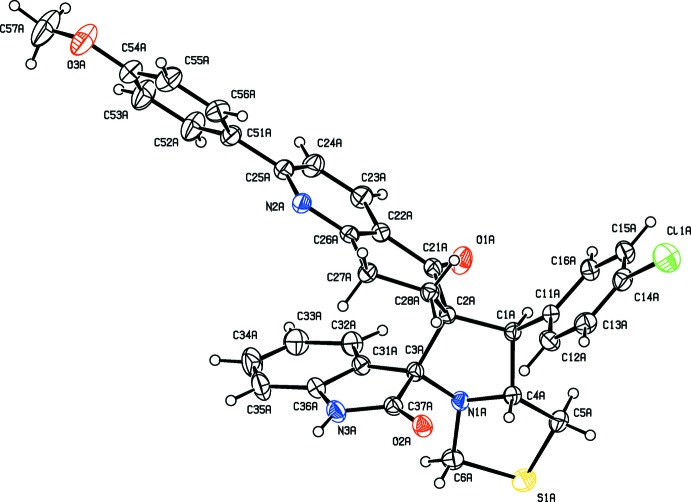
The mol­ecular structure of independent mol­ecule *A* of the title compound, showing 30% probability displacement ellipsoids and the atom labelling.

**Figure 3:**
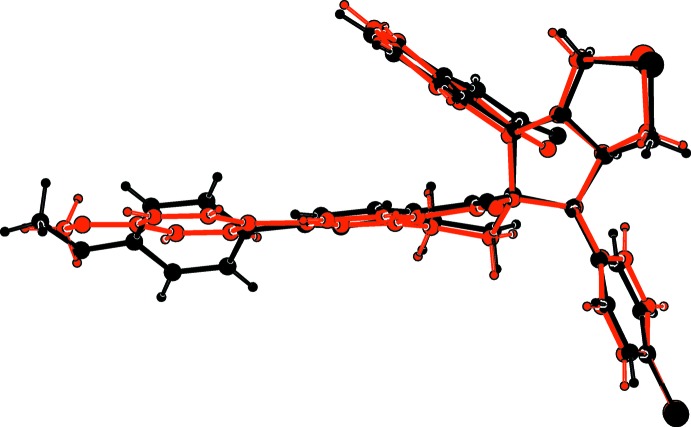
A view of the mol­ecular overlay of inverted mol­ecule *B* (red) on mol­ecule *A* (black).

The mol­ecular structure of the two independent mol­ecules, *A* and *B*, of the title compound are given in Figs. 1 ▸ and 2 ▸, respectively. The mol­ecular overlay of inverted mol­ecule *B* on mol­ecule *A* is shown in Fig. 3 ▸. The r.m.s. deviation is 0.44 Å with a maximum deviation of 1.931 Å (*Mercury*; Macrae *et al.*, 2008 ▸). The two mol­ecules differ essentially in the orientation of the 4-meth­oxy­phenyl ring (C51*A*–C56*A*, C51*B*–C56*B*) with respect to the pyridine ring of the iso­quinoline moiety (N2*A*/C22*A*–C26*A*, N2*B*/C22*B*–C26*B*). In mol­ecule A, the dihedral angle between the two rings is 37.01 (18)° compared to 7.06 (17)° in *B*. There is also a slight difference in the orientation of the 4-chloro­phenyl ring with respect to the mean plane of the pyrrolo ring, *viz*. in mol­ecule *A* benzene ring C11*A*–C16*A* is inclined to the mean plane of the pyrrol ring (N1*A*/C1*A*–C4*A*) by 86.12 (17)°, while in mol­ecule *B* the corresponding dihedral angle is 76.92 (17)°.

The cyclo­hexa­none ring [(C2*A*/C21*A*/C22*A*/C26*A*–C28*A*) in molecule *A* and (C2*B*/C21*B*/C22*B*/C26*B*–C28*B*) in *B*] of the iso­quinoline unit adopts a half-chair conformation in mol­ecule *A* [puckering parameters: amplitude *Q* = 0.441 (3) Å, θ = 50.1 (4)°, φ2 = 319.2 (6)°] and a distorted half-chair conformation in mol­ecule *B* [puckering parameters: amplitude *Q* = 0.502 (3) Å, θ = 123.5 (3)°, φ2 = 142.9 (5)°.

The thia­zole rings have twisted conformations on bonds C4*A*—N1*A* and C4*B*—N1*B* for mol­ecules *A* and *B*, respectively. The pyrrolo ring (N1*A*/C1*A*–C4*A*) has a twisted conformation on bond C4*A*–N1*A* in mol­ecule *A*, while in mol­ecule *B* this ring (N1*B*/C1*B*–C4*B*) has an envelope conformation with atom N1*B* as the flap. The mean planes of the thia­zole and pyrrolo rings are inclined to each other by 11.58 (17)° in *A* and 12.79 (18)° in *B*.

In the indolin-2-one ring systems [(N3*A*/C3*A*/C31*A*–C37*A*) in *A* and (N3*B*/C3*B*/C31*B*–C37*B*) in *B*], the benzene and pyrrolidine rings make dihedral angles of 3.65 (3) and 3.67 (3)° in mol­ecules *A* and *B*, respectively, while the keto atoms, O2*A* in mol­ecule *A* and O2*B* in mol­ecule *B*, deviate from the attached pyrrolidine rings by 0.1116 (1) and 0.0176 (1) Å, respectively.

As usual for such spiro compounds, the rings involving the spiro atoms (here C2*A*/C2*B* and C3*A*/C3*B*) are normal to each other. In mol­ecule *A*, the mean plane of the pyrrolo ring (N1*A*/C1*A*–C4*A*) is inclined to the mean planes of the pyrrolidine (N3*A*/C3*A*/C31*A*/C36*A*/C37*A*) and cyclo­hexa­none (C2*A*/C21*A*/C22*A*/C26*A*–C28*A*) rings by 86.14 (18) and 84.07 (12)°, respectively. In mol­ecule *B* the corresponding dihedral angles are 85.44 (18) and 85.34 (18)°.

## Supra­molecular features   

**Figure 4:**
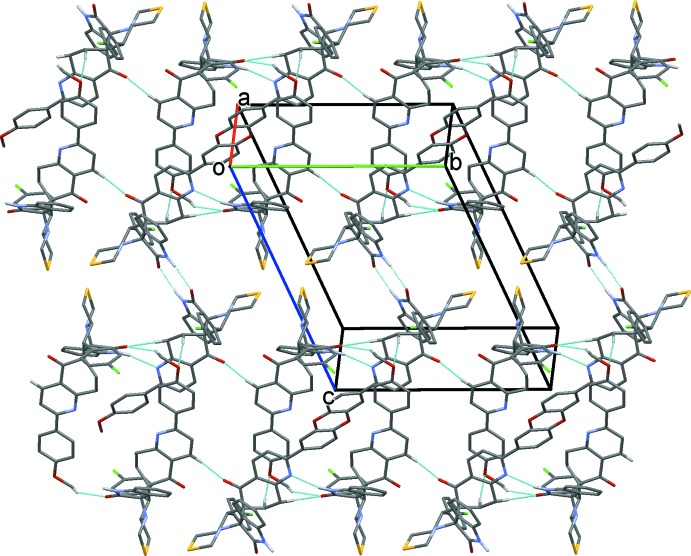
A view normal to plane (101) of the crystal packing of the title compound. The hydrogen bonds (see Table 1 ▸) are shown as dashed lines. H atoms not involved in these inter­actions have been omitted for clarity.

**Figure 5:**
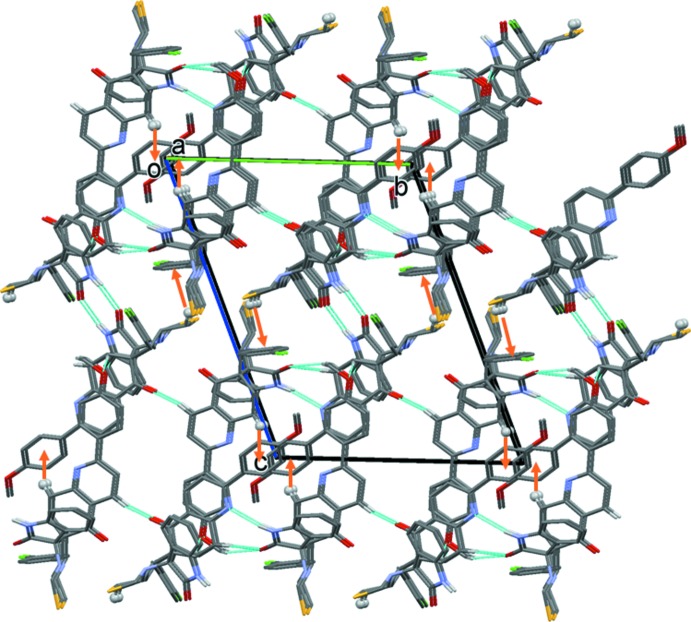
A view along the *a* axis of the crystal packing of the title compound. The hydrogen bonds are shown as dashed lines and the C—H⋯π inter­actions (Table 1 ▸) as orange arrows. H atoms not involved in these inter­actions have been omitted for clarity.

In the crystal, the *A* mol­ecules are linked by pairs of N—H⋯O hydrogen bonds, forming *A*–*A* inversion dimers with an 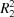
(8) ring motif (Fig. 4 ▸ and Table 1 ▸). These dimers are linked to the *B* mol­ecules by an N—H⋯N hydrogen bond and a series of C—H⋯O hydrogen bonds, forming layers lying parallel to the (101) plane. The layers are linked by C—H⋯π inter­actions (Fig. 5 ▸ and Table 1 ▸), and offset π–π inter­actions involving the pyridine ring (N2*A*/C22*A*–C26*A*; with centroid *Cg*4) of mol­ecule *A* and the 4-meth­oxy­phenyl ring (C51*B*–C56*B*; with centroid *Cg*19) of mol­ecule *B*: inter­centroid distance *Cg*4⋯*Cg*19 = 3.675 (2) Å, α = 7.84 (17)°, β = 13.3°, inter­planar distances = 3.427 (1) and 3.576 (1) Å, offset = 0.846 Å. These inter­actions lead to the formation of a supra­molecular framework (Fig. 5 ▸).

## Database survey   

A search of the Cambridge Structural Database (CSD, Version 5.39, last update August 2018; Groom *et al.*, 2016 ▸) for the central di­spiro fragment, 1′-methyl­dispiro­[cyclo­hexa­ne/cyclo­hexene-1,3′-pyrrolidine-2′,3′′-indoline]-2,2′′-dione, gave 16 hits (see supporting information file: CSD search.pdf). Three compounds closely resemble the title compound, *viz*. 4′-(4-chloro­phen­yl)-1′-methyl-3,4-di­hydro-1*H*-di­spiro­[acrid­ine-2,3′-pyrrolidine-2′,3′′-indole]-1,2′′(1′′*H*)-dione methanol solvate (CSD refcode NAQCAL; Maheswari *et al.*, 2012 ▸), 4′-(2,4-di­chloro­phen­yl)-10,3′′-dimethyl-1′′-phenyl-7′′,8′′-di­hydro­dispiro­[indole-3,2′-pyrrolidine-3′,6′′-pyrazolo­[3,4-*b*]quinoline]-2,5′′(1*H*,1′′*H*)-dione chloro­form solvate (UQIROD; Sumesh *et al.*, 2016 ▸), and 4′-(2-chloro­phen­yl)-1′-methyl-2′′-phenyl-7′′,8′′-di­hydro-5′′*H*-di­spiro­[indoline-3,2′-pyrrolidine-3′,6′′-quinoline]-2,5′′-dione (KEWKAB; Vishnupriya *et al.*, 2018 ▸). Three other compounds contain a pyrrolo­thia­zole moiety; they are the di­spiro­[cyclo­hexane-pyrrolo­thia­zole-indole]-dione derivatives RAGMUK, RAGNAR and RAHBIO (Lotfy *et al.*, 2017 ▸). In all six compounds, the mean plane of the pyrrolidine ring was found to be almost perpendicular to the mean plane of the indoline ring system and the mean plane of the cyclo­hexa­none ring, similar to the situation in the title compound.

## Synthesis and crystallization   

A mixture of isatin (1.1 mmol) and thia­zolidine-4-carb­oxy­lic acid (1.1 mmol) was taken in 10 ml of aceto­nitrile in a 50 ml round bottom flask and heated to reflux for 2 h. Then (*E*)-6-(4-chloro­benzyl­idene)-2-(4-meth­oxy­phen­yl)-7,8-di­hydro­quino­lin-5(6*H*)-one (1 mmol) was added and the reaction mixture was allowed to reflux for a further 14 h. After completion of the reaction, as evident from TLC, the solvent was removed under reduced pressure and the residue washed with ice-cold water (50 ml). The crude product was purified by column chromatography using 90:10 (*v*/*v*) petroleum ether–ethyl acetate mixtures to obtain the pure product. The product was dissolved in ethyl acetate and poured into a beaker, covered with perforated film and kept undisturbed. The solvent was allowed to evaporate slowly, yielding colourless block-like crystals after a period of seven days (m.p. 458 K; yield 80%).

## Refinement   

**Figure 6:**
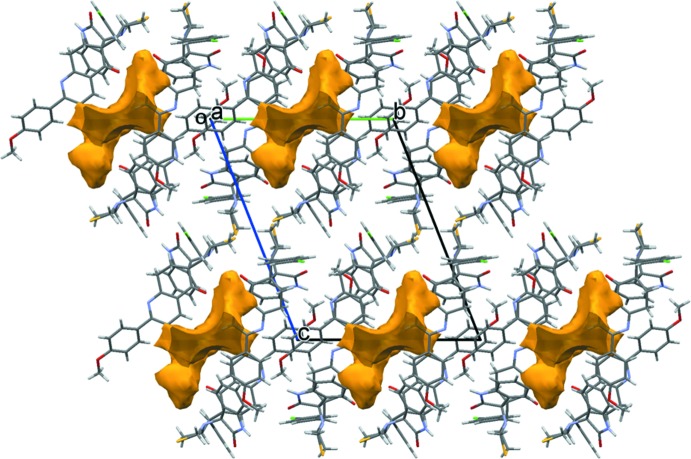
A view of the solvent-accessible surface (yellow/brown) in the crystal of the title compound.

Crystal data, data collection and structure refinement details are summarized in Table 2 ▸. The NH H atoms were located in a difference-Fourier map and freely refined. The C-bound H atoms were placed at calculated positions and allowed to ride on their carrier atoms: C—H = 0.93–0.98 Å with *U*_iso_ = 1.5*U*_eq_(C-meth­yl) and 1.2*U*_eq_(C) for other H atoms. The residual electron density was difficult to model and therefore, the SQUEEZE routine in *PLATON* (Spek, 2015 ▸) was used to remove the contribution of the electron density in the solvent region from the intensity data and the solvent-free model was employed for the final refinement. The solvent formula mass and unit-cell characteristics were not taken into account during refinement. The cavity of volume *ca* 418 Å^3^ (*ca* 14% of the unit-cell volume) contains approximately 100 electrons (see Fig. 6 ▸).

## Supplementary Material

Crystal structure: contains datablock(s) global, I. DOI: 10.1107/S2056989019000112/su5469sup1.cif

Structure factors: contains datablock(s) I. DOI: 10.1107/S2056989019000112/su5469Isup2.hkl

CSD search. DOI: 10.1107/S2056989019000112/su5469sup3.pdf

CCDC reference: 1888600

Additional supporting information:  crystallographic information; 3D view; checkCIF report

## Figures and Tables

**Figure 2:**
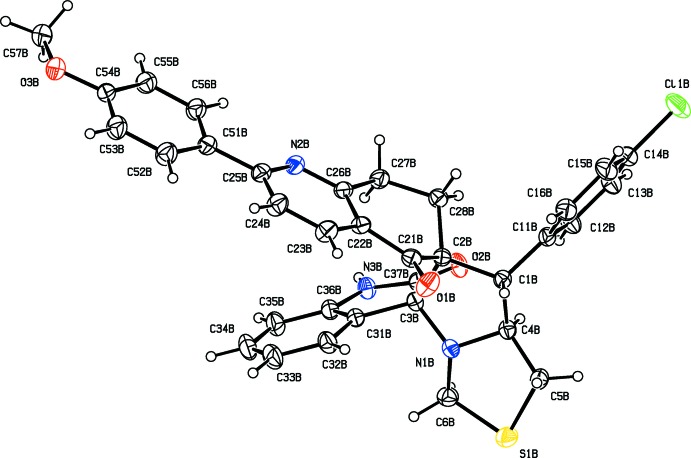
The mol­ecular structure of independent mol­ecule *B* of the title compound, showing 30% probability displacement ellipsoids and the atom labelling.

**Table 1:** Hydrogen-bond geometry (Å, °) *Cg*8 and *Cg*17 are the centroids of the C51*A*–C56*A* and C11*B*–C16*B* rings, respectively.

*D*—H⋯*A*	*D*—H	H⋯*A*	*D*⋯*A*	*D*—H⋯*A*
N3*A*—H3*A*⋯O2*A* ^i^	0.83 (4)	2.05 (4)	2.874 (4)	172 (4)
N3*B*—H3*B*⋯N2*A* ^ii^	0.91 (4)	2.24 (4)	3.136 (4)	169 (3)
C23*B*—H23*B*⋯O1*A*	0.93	2.36	3.066 (4)	132
C27*A*—H27*D*⋯O2*B* ^iii^	0.97	2.40	3.371 (4)	177
C28*A*—H28*C*⋯O3*B* ^iv^	0.97	2.55	3.517 (4)	175
C57*B*—H57*C*⋯O2*B* ^v^	0.96	2.50	3.349 (5)	147
C5*A*—H5*A*1⋯*Cg*17^vi^	0.97	2.91	3.711 (4)	140
C27*B*—H27*A*⋯*Cg*8^iv^	0.97	2.89	3.783 (4)	154

Symmetry codes: (i) 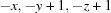; (ii) 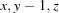; (iii) 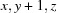; (iv) 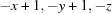; (v) 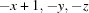; (vi) 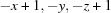.

**Table 2:** Experimental details

Crystal data	
Chemical formula	C_34_H_28_ClN_3_O_3_S
*M*_r_	594.10
Crystal system, space group	Triclinic, *P* 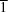
Temperature (K)	293
*a*, *b*, *c* (Å)	11.8222 (7), 14.7535 (9), 19.5055 (12)
α, β, γ (°)	68.396 (3), 78.555 (3), 87.302 (3)
*V* (Å^3^)	3099.0 (3)
*Z*	4
Radiation type	Mo *K*α
μ (mm^−1^)	0.23
Crystal size (mm)	0.21 × 0.2 × 0.18

Data collection	
Diffractometer	Bruker Kappa APEXII
Absorption correction	Multi-scan (*SADABS*; Bruker, 2009 ▸)
*T*_min_, *T*_max_	0.967, 0.974
No. of measured, independent and observed [*I* > 2σ(*I*)] reflections	78936, 12885, 7510
*R*_int_	0.072
(sin θ/λ)_max_ (Å^−1^)	0.630

Refinement	
*R*[*F* ^2^ > 2σ(*F* ^2^)], *wR*(*F* ^2^), *S*	0.058, 0.171, 1.01
No. of reflections	12885
No. of parameters	765
H-atom treatment	H atoms treated by a mixture of independent and constrained refinement
Δρ_max_, Δρ_min_ (e Å^−3^)	0.40, −0.32

Computer programs: *APEX2* and *SAINT* (Bruker, 2009 ▸), *SHELXS2013* (Sheldrick, 2008 ▸), *SHELXL2014/6* (Sheldrick, 2015 ▸), *ORTEP-3 for Windows* (Farrugia, 2012 ▸), *Mercury* (Macrae *et al.*, 2008 ▸), *PLATON* (Spek, 2009 ▸) and *publCIF* (Westrip, 2010 ▸).

## supplementary crystallographic information

### Crystal data

**Table:**

C_34_H_28_ClN_3_O_3_S	*Z* = 4
*M**_r_* = 594.10	*F*(000) = 1240
Triclinic, *P*1	*D*_x_ = 1.273 Mg m^−^^3^
*a* = 11.8222 (7) Å	Mo *K*α radiation, λ = 0.71073 Å
*b* = 14.7535 (9) Å	Cell parameters from 12885 reflections
*c* = 19.5055 (12) Å	θ = 2.2–26.6°
α = 68.396 (3)°	µ = 0.23 mm^−^^1^
β = 78.555 (3)°	*T* = 293 K
γ = 87.302 (3)°	Block, colourless
*V* = 3099.0 (3) Å^3^	0.21 × 0.2 × 0.18 mm

### Data collection

**Table:**

Bruker Kappa APEXII diffractometer	12885 independent reflections
Radiation source: fine-focus sealed tube	7510 reflections with *I* > 2σ(*I*)
Detector resolution: 0 pixels mm^-1^	*R*_int_ = 0.072
ω and φ scans	θ_max_ = 26.6°, θ_min_ = 2.2°
Absorption correction: multi-scan (SADABS; Bruker, 2009)	*h* = −14→14
*T*_min_ = 0.967, *T*_max_ = 0.974	*k* = −18→18
78936 measured reflections	*l* = −24→24

### Refinement

**Table:**

Refinement on *F*^2^	Primary atom site location: structure-invariant direct methods
Least-squares matrix: full	Secondary atom site location: difference Fourier map
*R*[*F*^2^ > 2σ(*F*^2^)] = 0.058	Hydrogen site location: difference Fourier map
*wR*(*F*^2^) = 0.171	H atoms treated by a mixture of independent and constrained refinement
*S* = 1.01	*w* = 1/[σ^2^(*F*_o_^2^) + (0.067*P*)^2^ + 2.7018*P*] where *P* = (*F*_o_^2^ + 2*F*_c_^2^)/3
12885 reflections	(Δ/σ)_max_ = 0.001
765 parameters	Δρ_max_ = 0.40 e Å^−^^3^
0 restraints	Δρ_min_ = −0.32 e Å^−^^3^

### Special details

**Table:**

Geometry. All esds (except the esd in the dihedral angle between two l.s. planes) are estimated using the full covariance matrix. The cell esds are taken into account individually in the estimation of esds in distances, angles and torsion angles; correlations between esds in cell parameters are only used when they are defined by crystal symmetry. An approximate (isotropic) treatment of cell esds is used for estimating esds involving l.s. planes.

### Fractional atomic coordinates and isotropic or equivalent isotropic displacement parameters (Å^2^)

**Table:**

	*x*	*y*	*z*	*U*_iso_*/*U*_eq_	
C1B	0.5260 (2)	−0.0249 (2)	0.35951 (16)	0.0323 (7)	
H1B	0.5318	0.0323	0.3729	0.039*	
C1A	0.3327 (2)	0.3432 (2)	0.36470 (16)	0.0308 (6)	
H1A	0.3600	0.3148	0.3264	0.037*	
C2B	0.4873 (3)	0.0134 (2)	0.28085 (16)	0.0321 (7)	
C2A	0.2713 (2)	0.44096 (19)	0.32533 (15)	0.0284 (6)	
C3B	0.3566 (2)	−0.0264 (2)	0.30098 (16)	0.0312 (6)	
C3A	0.1371 (2)	0.4122 (2)	0.35761 (16)	0.0320 (7)	
C4B	0.4242 (2)	−0.0873 (2)	0.41544 (16)	0.0341 (7)	
H4B	0.4308	−0.1549	0.4179	0.041*	
C4A	0.2363 (2)	0.2758 (2)	0.42160 (16)	0.0326 (7)	
H4A	0.2214	0.2894	0.4681	0.039*	
C5B	0.3932 (3)	−0.0848 (3)	0.49356 (17)	0.0428 (8)	
H5A	0.4114	−0.0211	0.4932	0.051*	
H5B	0.4345	−0.1337	0.5275	0.051*	
C5A	0.2420 (3)	0.1669 (2)	0.43995 (19)	0.0426 (8)	
H5A1	0.2781	0.1356	0.4831	0.051*	
H5A2	0.2854	0.1527	0.3975	0.051*	
C6B	0.2225 (3)	−0.0925 (2)	0.42665 (17)	0.0426 (8)	
H6A	0.2145	−0.1544	0.4210	0.051*	
H6B	0.1558	−0.0538	0.4139	0.051*	
C6A	0.0382 (3)	0.2480 (2)	0.4275 (2)	0.0464 (8)	
H6A1	−0.0193	0.2527	0.3972	0.056*	
H6A2	0.0046	0.2678	0.4692	0.056*	
C11B	0.6425 (3)	−0.0712 (2)	0.36006 (16)	0.0345 (7)	
C11A	0.4363 (2)	0.3534 (2)	0.39620 (16)	0.0314 (6)	
C12B	0.6578 (3)	−0.1687 (2)	0.37073 (19)	0.0439 (8)	
H12B	0.5939	−0.2084	0.3787	0.053*	
C12A	0.4272 (3)	0.3740 (2)	0.46068 (18)	0.0421 (8)	
H12A	0.3546	0.3823	0.4862	0.050*	
C13B	0.7665 (3)	−0.2084 (2)	0.36978 (19)	0.0474 (8)	
H13B	0.7751	−0.2743	0.3776	0.057*	
C13A	0.5241 (3)	0.3824 (2)	0.48810 (18)	0.0443 (8)	
H13A	0.5167	0.3964	0.5315	0.053*	
C14B	0.8603 (3)	−0.1508 (3)	0.35735 (18)	0.0471 (8)	
C14A	0.6301 (3)	0.3699 (2)	0.45095 (19)	0.0422 (8)	
C15B	0.8488 (3)	−0.0547 (3)	0.3474 (2)	0.0525 (9)	
H15B	0.9134	−0.0158	0.3396	0.063*	
C15A	0.6430 (3)	0.3497 (2)	0.3871 (2)	0.0461 (8)	
H15A	0.7162	0.3422	0.3618	0.055*	
C16B	0.7397 (3)	−0.0152 (2)	0.34917 (18)	0.0450 (8)	
H16B	0.7318	0.0502	0.3429	0.054*	
C16A	0.5461 (3)	0.3405 (2)	0.36039 (18)	0.0387 (7)	
H16A	0.5546	0.3252	0.3175	0.046*	
C21B	0.4888 (3)	0.1254 (2)	0.25042 (17)	0.0359 (7)	
C21A	0.2926 (3)	0.4648 (2)	0.24032 (16)	0.0345 (7)	
C22B	0.4879 (3)	0.1759 (2)	0.16965 (16)	0.0354 (7)	
C22A	0.2763 (3)	0.5667 (2)	0.18981 (16)	0.0345 (7)	
C23B	0.4699 (3)	0.2756 (2)	0.14136 (18)	0.0465 (8)	
H23B	0.4663	0.3118	0.1719	0.056*	
C23A	0.2758 (3)	0.5862 (2)	0.11482 (18)	0.0459 (8)	
H23A	0.2877	0.5364	0.0959	0.055*	
C24B	0.4577 (3)	0.3198 (2)	0.06846 (18)	0.0491 (9)	
H24B	0.4470	0.3867	0.0489	0.059*	
C24A	0.2579 (3)	0.6787 (3)	0.06877 (19)	0.0488 (9)	
H24A	0.2563	0.6924	0.0185	0.059*	
C25B	0.4613 (3)	0.2653 (2)	0.02361 (17)	0.0407 (8)	
C25A	0.2422 (3)	0.7517 (2)	0.09796 (18)	0.0409 (8)	
C26B	0.4963 (3)	0.1263 (2)	0.12049 (16)	0.0338 (7)	
C26A	0.2602 (2)	0.6436 (2)	0.21523 (16)	0.0340 (7)	
C27B	0.5222 (3)	0.0205 (2)	0.14588 (17)	0.0393 (7)	
H27A	0.5808	0.0091	0.1076	0.047*	
H27B	0.4530	−0.0165	0.1515	0.047*	
C27A	0.2574 (3)	0.6260 (2)	0.29575 (16)	0.0361 (7)	
H27C	0.1779	0.6257	0.3209	0.043*	
H27D	0.2984	0.6794	0.2989	0.043*	
C28B	0.5642 (3)	−0.0159 (2)	0.21994 (16)	0.0367 (7)	
H28A	0.6419	0.0098	0.2114	0.044*	
H28B	0.5675	−0.0865	0.2378	0.044*	
C28A	0.3113 (3)	0.5301 (2)	0.33682 (16)	0.0329 (7)	
H28C	0.3945	0.5375	0.3204	0.040*	
H28D	0.2941	0.5181	0.3902	0.040*	
C31B	0.2667 (3)	0.0329 (2)	0.26030 (16)	0.0343 (7)	
C31A	0.0542 (3)	0.4546 (2)	0.30558 (17)	0.0353 (7)	
C32B	0.2235 (3)	0.1228 (2)	0.25610 (19)	0.0447 (8)	
H32B	0.2546	0.1598	0.2778	0.054*	
C32A	0.0418 (3)	0.4413 (2)	0.24147 (19)	0.0463 (8)	
H32A	0.0889	0.3991	0.2239	0.056*	
C33B	0.1324 (3)	0.1571 (3)	0.2188 (2)	0.0576 (10)	
H33B	0.1031	0.2181	0.2147	0.069*	
C33A	−0.0430 (3)	0.4924 (3)	0.2033 (2)	0.0586 (10)	
H33A	−0.0522	0.4848	0.1594	0.070*	
C34B	0.0855 (3)	0.1012 (3)	0.1878 (2)	0.0591 (10)	
H34B	0.0253	0.1256	0.1625	0.071*	
C34A	−0.1127 (3)	0.5535 (3)	0.2300 (2)	0.0685 (12)	
H34A	−0.1675	0.5885	0.2031	0.082*	
C35B	0.1254 (3)	0.0101 (3)	0.1932 (2)	0.0501 (9)	
H35B	0.0927	−0.0279	0.1728	0.060*	
C35A	−0.1038 (3)	0.5645 (3)	0.2956 (2)	0.0620 (11)	
H35A	−0.1526	0.6052	0.3139	0.074*	
C36B	0.2163 (3)	−0.0223 (2)	0.23029 (17)	0.0370 (7)	
C36A	−0.0204 (3)	0.5134 (2)	0.33343 (18)	0.0396 (7)	
C37B	0.3501 (3)	−0.1224 (2)	0.28654 (16)	0.0342 (7)	
C37A	0.0894 (3)	0.4501 (2)	0.42181 (17)	0.0330 (7)	
C51B	0.4430 (3)	0.3075 (2)	−0.05451 (17)	0.0391 (7)	
C51A	0.2227 (3)	0.8532 (2)	0.04837 (18)	0.0434 (8)	
C52B	0.4088 (3)	0.4027 (3)	−0.0863 (2)	0.0526 (9)	
H52B	0.3966	0.4420	−0.0578	0.063*	
C52A	0.1596 (4)	0.8708 (3)	−0.0074 (2)	0.0645 (11)	
H52A	0.1286	0.8181	−0.0135	0.077*	
C53B	0.3926 (3)	0.4398 (3)	−0.1591 (2)	0.0526 (9)	
H53B	0.3707	0.5041	−0.1797	0.063*	
C53A	0.1407 (4)	0.9640 (3)	−0.0545 (2)	0.0702 (12)	
H53A	0.0978	0.9734	−0.0916	0.084*	
C54B	0.4085 (3)	0.3825 (2)	−0.20156 (17)	0.0407 (8)	
C54A	0.1851 (3)	1.0421 (3)	−0.0465 (2)	0.0586 (10)	
C55B	0.4435 (3)	0.2881 (2)	−0.17192 (19)	0.0473 (8)	
H55B	0.4561	0.2493	−0.2009	0.057*	
C55A	0.2505 (4)	1.0263 (3)	0.0077 (2)	0.0597 (10)	
H55A	0.2830	1.0791	0.0127	0.072*	
C56B	0.4595 (3)	0.2517 (2)	−0.09857 (18)	0.0468 (8)	
H56B	0.4821	0.1876	−0.0783	0.056*	
C56A	0.2681 (3)	0.9333 (3)	0.05454 (19)	0.0511 (9)	
H56A	0.3117	0.9243	0.0912	0.061*	
N1B	0.3275 (2)	−0.04168 (17)	0.38060 (13)	0.0329 (6)	
N1A	0.1396 (2)	0.30634 (17)	0.38312 (13)	0.0336 (6)	
N2B	0.4825 (2)	0.16914 (18)	0.04989 (14)	0.0394 (6)	
N2A	0.2435 (2)	0.73521 (18)	0.17025 (14)	0.0389 (6)	
N3B	0.2667 (2)	−0.11384 (19)	0.24572 (15)	0.0378 (6)	
N3A	0.0019 (2)	0.5097 (2)	0.40164 (16)	0.0423 (7)	
O1B	0.4912 (2)	0.17151 (15)	0.28999 (12)	0.0506 (6)	
O1A	0.3175 (2)	0.40314 (17)	0.21365 (12)	0.0573 (7)	
O2B	0.40684 (19)	−0.19446 (15)	0.30953 (13)	0.0441 (5)	
O2A	0.12263 (18)	0.42887 (15)	0.48137 (12)	0.0412 (5)	
O3B	0.3894 (2)	0.42566 (17)	−0.27308 (12)	0.0518 (6)	
O3A	0.1723 (3)	1.13645 (19)	−0.09003 (17)	0.0847 (10)	
S1B	0.23841 (8)	−0.11082 (7)	0.52141 (5)	0.0504 (2)	
S1A	0.09202 (9)	0.12520 (6)	0.46069 (6)	0.0564 (3)	
Cl1B	0.99560 (8)	−0.20181 (9)	0.35523 (7)	0.0754 (3)	
Cl1A	0.75107 (9)	0.38243 (8)	0.48536 (6)	0.0711 (3)	
C57B	0.3971 (3)	0.3675 (3)	−0.3168 (2)	0.0545 (9)	
H57A	0.3819	0.4063	−0.3656	0.082*	
H57C	0.4732	0.3421	−0.3221	0.082*	
H57B	0.3413	0.3146	−0.2927	0.082*	
C57A	0.1111 (5)	1.1555 (4)	−0.1488 (3)	0.112 (2)	
H57D	0.1086	1.2246	−0.1749	0.168*	
H57E	0.1492	1.1259	−0.1833	0.168*	
H57F	0.0338	1.1288	−0.1283	0.168*	
H3B	0.251 (3)	−0.161 (3)	0.229 (2)	0.063 (12)*	
H3A	−0.037 (4)	0.532 (3)	0.432 (2)	0.074 (14)*	

### Atomic displacement parameters (Å^2^)

**Table:**

	*U*^11^	*U*^22^	*U*^33^	*U*^12^	*U*^13^	*U*^23^
C1B	0.0321 (16)	0.0331 (15)	0.0321 (16)	−0.0013 (13)	−0.0072 (13)	−0.0117 (13)
C1A	0.0332 (16)	0.0284 (15)	0.0301 (15)	0.0057 (12)	−0.0062 (13)	−0.0105 (12)
C2B	0.0333 (16)	0.0323 (15)	0.0322 (16)	0.0036 (13)	−0.0071 (13)	−0.0136 (13)
C2A	0.0255 (15)	0.0279 (14)	0.0319 (15)	0.0032 (12)	−0.0055 (12)	−0.0115 (12)
C3B	0.0300 (16)	0.0305 (15)	0.0361 (16)	0.0050 (12)	−0.0112 (13)	−0.0138 (13)
C3A	0.0325 (16)	0.0281 (15)	0.0367 (16)	0.0046 (12)	−0.0059 (13)	−0.0141 (13)
C4B	0.0326 (16)	0.0326 (16)	0.0364 (17)	0.0001 (13)	−0.0100 (13)	−0.0100 (13)
C4A	0.0331 (16)	0.0301 (15)	0.0340 (16)	0.0033 (13)	−0.0053 (13)	−0.0119 (13)
C5B	0.0398 (19)	0.052 (2)	0.0340 (17)	−0.0041 (15)	−0.0109 (15)	−0.0099 (15)
C5A	0.048 (2)	0.0299 (16)	0.0466 (19)	0.0050 (14)	−0.0085 (16)	−0.0112 (14)
C6B	0.0390 (19)	0.0427 (18)	0.0428 (19)	−0.0013 (15)	−0.0031 (15)	−0.0140 (15)
C6A	0.0373 (19)	0.0365 (17)	0.060 (2)	−0.0059 (14)	−0.0059 (16)	−0.0130 (16)
C11B	0.0303 (16)	0.0394 (17)	0.0325 (16)	−0.0012 (13)	−0.0086 (13)	−0.0100 (13)
C11A	0.0284 (16)	0.0301 (15)	0.0329 (16)	0.0039 (12)	−0.0060 (13)	−0.0086 (13)
C12B	0.0315 (17)	0.0454 (19)	0.057 (2)	−0.0010 (14)	−0.0123 (15)	−0.0185 (16)
C12A	0.0315 (17)	0.052 (2)	0.0418 (19)	0.0042 (15)	−0.0043 (14)	−0.0177 (16)
C13B	0.041 (2)	0.0433 (19)	0.057 (2)	0.0096 (16)	−0.0143 (17)	−0.0164 (17)
C13A	0.048 (2)	0.0467 (19)	0.0387 (18)	−0.0006 (16)	−0.0112 (16)	−0.0143 (15)
C14B	0.0302 (18)	0.062 (2)	0.0420 (19)	0.0042 (16)	−0.0066 (15)	−0.0117 (17)
C14A	0.0399 (19)	0.0363 (17)	0.050 (2)	−0.0017 (14)	−0.0193 (16)	−0.0099 (15)
C15B	0.0294 (18)	0.063 (2)	0.059 (2)	−0.0086 (16)	−0.0085 (16)	−0.0143 (19)
C15A	0.0334 (18)	0.0461 (19)	0.057 (2)	0.0070 (15)	−0.0095 (16)	−0.0177 (17)
C16B	0.0388 (19)	0.0455 (19)	0.047 (2)	−0.0065 (15)	−0.0089 (16)	−0.0112 (16)
C16A	0.0363 (18)	0.0392 (17)	0.0418 (18)	0.0067 (14)	−0.0083 (15)	−0.0165 (14)
C21B	0.0370 (17)	0.0340 (16)	0.0394 (18)	0.0028 (13)	−0.0059 (14)	−0.0176 (14)
C21A	0.0331 (17)	0.0358 (16)	0.0345 (17)	0.0053 (13)	−0.0041 (13)	−0.0147 (14)
C22B	0.0373 (17)	0.0338 (16)	0.0323 (16)	0.0001 (13)	−0.0014 (13)	−0.0116 (13)
C22A	0.0332 (17)	0.0362 (16)	0.0327 (16)	0.0032 (13)	−0.0060 (13)	−0.0113 (13)
C23B	0.062 (2)	0.0357 (17)	0.0421 (19)	0.0043 (16)	−0.0073 (17)	−0.0164 (15)
C23A	0.053 (2)	0.0440 (19)	0.0404 (19)	0.0042 (16)	−0.0071 (16)	−0.0170 (16)
C24B	0.068 (2)	0.0322 (17)	0.041 (2)	0.0078 (16)	−0.0086 (17)	−0.0084 (15)
C24A	0.056 (2)	0.053 (2)	0.0357 (18)	0.0036 (17)	−0.0158 (17)	−0.0101 (16)
C25B	0.0413 (19)	0.0405 (18)	0.0342 (17)	0.0030 (15)	−0.0029 (14)	−0.0092 (14)
C25A	0.0369 (18)	0.0405 (18)	0.0396 (19)	0.0002 (14)	−0.0086 (15)	−0.0072 (15)
C26B	0.0331 (16)	0.0355 (16)	0.0313 (16)	0.0015 (13)	−0.0026 (13)	−0.0125 (13)
C26A	0.0258 (15)	0.0376 (17)	0.0354 (17)	−0.0021 (13)	−0.0037 (13)	−0.0104 (14)
C27B	0.0460 (19)	0.0384 (17)	0.0358 (17)	0.0068 (15)	−0.0077 (15)	−0.0170 (14)
C27A	0.0384 (18)	0.0273 (15)	0.0410 (18)	0.0000 (13)	−0.0068 (14)	−0.0110 (13)
C28B	0.0383 (18)	0.0347 (16)	0.0373 (17)	0.0037 (14)	−0.0066 (14)	−0.0142 (14)
C28A	0.0322 (16)	0.0338 (16)	0.0335 (16)	0.0012 (13)	−0.0082 (13)	−0.0123 (13)
C31B	0.0326 (17)	0.0379 (17)	0.0339 (16)	0.0051 (13)	−0.0093 (13)	−0.0139 (13)
C31A	0.0300 (16)	0.0375 (17)	0.0383 (17)	0.0006 (13)	−0.0101 (14)	−0.0119 (14)
C32B	0.043 (2)	0.0434 (19)	0.052 (2)	0.0129 (15)	−0.0165 (16)	−0.0202 (16)
C32A	0.046 (2)	0.048 (2)	0.050 (2)	0.0043 (16)	−0.0157 (17)	−0.0213 (16)
C33B	0.058 (2)	0.051 (2)	0.065 (2)	0.0263 (19)	−0.023 (2)	−0.0205 (19)
C33A	0.053 (2)	0.073 (3)	0.054 (2)	−0.001 (2)	−0.0235 (19)	−0.022 (2)
C34B	0.048 (2)	0.067 (3)	0.065 (3)	0.0194 (19)	−0.0293 (19)	−0.021 (2)
C34A	0.049 (2)	0.089 (3)	0.068 (3)	0.024 (2)	−0.031 (2)	−0.023 (2)
C35B	0.044 (2)	0.058 (2)	0.055 (2)	0.0074 (17)	−0.0232 (17)	−0.0214 (18)
C35A	0.044 (2)	0.079 (3)	0.067 (3)	0.029 (2)	−0.0198 (19)	−0.030 (2)
C36B	0.0349 (17)	0.0376 (17)	0.0379 (17)	0.0036 (14)	−0.0114 (14)	−0.0113 (14)
C36A	0.0311 (17)	0.0419 (18)	0.0435 (19)	0.0067 (14)	−0.0054 (14)	−0.0146 (15)
C37B	0.0335 (17)	0.0344 (16)	0.0352 (17)	0.0041 (13)	−0.0099 (14)	−0.0120 (13)
C37A	0.0306 (16)	0.0316 (15)	0.0371 (17)	0.0005 (13)	−0.0039 (14)	−0.0142 (13)
C51B	0.0400 (18)	0.0371 (17)	0.0349 (17)	0.0017 (14)	−0.0053 (14)	−0.0085 (14)
C51A	0.045 (2)	0.0366 (17)	0.0416 (19)	−0.0016 (15)	−0.0101 (16)	−0.0054 (15)
C52B	0.065 (2)	0.046 (2)	0.046 (2)	0.0096 (18)	−0.0132 (18)	−0.0164 (17)
C52A	0.076 (3)	0.046 (2)	0.071 (3)	−0.001 (2)	−0.040 (2)	−0.0081 (19)
C53B	0.062 (2)	0.0424 (19)	0.048 (2)	0.0092 (17)	−0.0147 (18)	−0.0086 (17)
C53A	0.082 (3)	0.056 (2)	0.069 (3)	0.004 (2)	−0.042 (2)	−0.005 (2)
C54B	0.0330 (17)	0.0470 (19)	0.0338 (17)	0.0016 (14)	−0.0051 (14)	−0.0060 (15)
C54A	0.063 (3)	0.047 (2)	0.049 (2)	0.0032 (19)	−0.0100 (19)	0.0007 (18)
C55B	0.052 (2)	0.0453 (19)	0.044 (2)	0.0076 (16)	−0.0097 (16)	−0.0155 (16)
C55A	0.078 (3)	0.044 (2)	0.053 (2)	−0.0039 (19)	−0.011 (2)	−0.0130 (18)
C56B	0.055 (2)	0.0403 (18)	0.0395 (19)	0.0086 (16)	−0.0094 (16)	−0.0087 (15)
C56A	0.058 (2)	0.052 (2)	0.0384 (19)	−0.0020 (18)	−0.0086 (17)	−0.0116 (16)
N1B	0.0287 (13)	0.0336 (13)	0.0365 (14)	0.0015 (11)	−0.0087 (11)	−0.0121 (11)
N1A	0.0303 (14)	0.0285 (13)	0.0409 (14)	0.0016 (10)	−0.0077 (11)	−0.0112 (11)
N2B	0.0431 (16)	0.0391 (15)	0.0322 (14)	0.0039 (12)	−0.0059 (12)	−0.0098 (12)
N2A	0.0345 (15)	0.0352 (14)	0.0419 (16)	−0.0001 (11)	−0.0102 (12)	−0.0066 (12)
N3B	0.0388 (15)	0.0352 (14)	0.0447 (16)	0.0011 (12)	−0.0164 (13)	−0.0166 (12)
N3A	0.0369 (16)	0.0492 (17)	0.0423 (17)	0.0120 (13)	−0.0048 (13)	−0.0215 (14)
O1B	0.0823 (18)	0.0331 (12)	0.0411 (13)	0.0022 (12)	−0.0141 (12)	−0.0178 (10)
O1A	0.0852 (19)	0.0486 (14)	0.0425 (14)	0.0285 (13)	−0.0128 (13)	−0.0248 (12)
O2B	0.0461 (14)	0.0334 (12)	0.0592 (15)	0.0087 (10)	−0.0226 (11)	−0.0192 (11)
O2A	0.0410 (13)	0.0474 (13)	0.0384 (13)	0.0095 (10)	−0.0080 (10)	−0.0201 (10)
O3B	0.0545 (15)	0.0529 (14)	0.0413 (13)	0.0095 (12)	−0.0129 (11)	−0.0086 (11)
O3A	0.111 (3)	0.0476 (16)	0.081 (2)	0.0039 (16)	−0.0381 (19)	0.0030 (15)
S1B	0.0416 (5)	0.0637 (6)	0.0402 (5)	−0.0072 (4)	−0.0027 (4)	−0.0140 (4)
S1A	0.0539 (6)	0.0348 (5)	0.0721 (7)	−0.0071 (4)	−0.0061 (5)	−0.0121 (4)
Cl1B	0.0347 (5)	0.0922 (8)	0.0890 (8)	0.0150 (5)	−0.0113 (5)	−0.0234 (6)
Cl1A	0.0504 (6)	0.0867 (7)	0.0837 (7)	−0.0068 (5)	−0.0301 (5)	−0.0301 (6)
C57B	0.051 (2)	0.070 (2)	0.046 (2)	0.0097 (19)	−0.0162 (17)	−0.0227 (19)
C57A	0.151 (6)	0.072 (3)	0.091 (4)	0.016 (3)	−0.061 (4)	0.013 (3)

### Geometric parameters (Å, º)

**Table:**

C1B—C11B	1.509 (4)	C24A—H24A	0.9300
C1B—C4B	1.526 (4)	C25B—N2B	1.349 (4)
C1B—C2B	1.580 (4)	C25B—C51B	1.475 (4)
C1B—H1B	0.9800	C25A—N2A	1.343 (4)
C1A—C11A	1.512 (4)	C25A—C51A	1.490 (4)
C1A—C4A	1.521 (4)	C26B—N2B	1.327 (4)
C1A—C2A	1.582 (4)	C26B—C27B	1.491 (4)
C1A—H1A	0.9800	C26A—N2A	1.344 (4)
C2B—C28B	1.529 (4)	C26A—C27A	1.489 (4)
C2B—C21B	1.535 (4)	C27B—C28B	1.520 (4)
C2B—C3B	1.601 (4)	C27B—H27A	0.9700
C2A—C28A	1.523 (4)	C27B—H27B	0.9700
C2A—C21A	1.533 (4)	C27A—C28A	1.527 (4)
C2A—C3A	1.601 (4)	C27A—H27C	0.9700
C3B—N1B	1.455 (4)	C27A—H27D	0.9700
C3B—C31B	1.503 (4)	C28B—H28A	0.9700
C3B—C37B	1.550 (4)	C28B—H28B	0.9700
C3A—N1A	1.455 (3)	C28A—H28C	0.9700
C3A—C31A	1.501 (4)	C28A—H28D	0.9700
C3A—C37A	1.551 (4)	C31B—C36B	1.377 (4)
C4B—N1B	1.457 (4)	C31B—C32B	1.379 (4)
C4B—C5B	1.509 (4)	C31A—C32A	1.371 (4)
C4B—H4B	0.9800	C31A—C36A	1.381 (4)
C4A—N1A	1.454 (4)	C32B—C33B	1.391 (4)
C4A—C5A	1.513 (4)	C32B—H32B	0.9300
C4A—H4A	0.9800	C32A—C33A	1.392 (5)
C5B—S1B	1.819 (3)	C32A—H32A	0.9300
C5B—H5A	0.9700	C33B—C34B	1.378 (5)
C5B—H5B	0.9700	C33B—H33B	0.9300
C5A—S1A	1.822 (3)	C33A—C34A	1.368 (5)
C5A—H5A1	0.9700	C33A—H33A	0.9300
C5A—H5A2	0.9700	C34B—C35B	1.377 (5)
C6B—N1B	1.435 (4)	C34B—H34B	0.9300
C6B—S1B	1.814 (3)	C34A—C35A	1.371 (5)
C6B—H6A	0.9700	C34A—H34A	0.9300
C6B—H6B	0.9700	C35B—C36B	1.383 (4)
C6A—N1A	1.437 (4)	C35B—H35B	0.9300
C6A—S1A	1.815 (3)	C35A—C36A	1.376 (4)
C6A—H6A1	0.9700	C35A—H35A	0.9300
C6A—H6A2	0.9700	C36B—N3B	1.401 (4)
C11B—C16B	1.383 (4)	C36A—N3A	1.389 (4)
C11B—C12B	1.384 (4)	C37B—O2B	1.218 (3)
C11A—C12A	1.382 (4)	C37B—N3B	1.360 (4)
C11A—C16A	1.387 (4)	C37A—O2A	1.226 (3)
C12B—C13B	1.387 (4)	C37A—N3A	1.348 (4)
C12B—H12B	0.9300	C51B—C56B	1.377 (4)
C12A—C13A	1.385 (4)	C51B—C52B	1.387 (4)
C12A—H12A	0.9300	C51A—C56A	1.374 (5)
C13B—C14B	1.357 (5)	C51A—C52A	1.380 (5)
C13B—H13B	0.9300	C52B—C53B	1.370 (5)
C13A—C14A	1.358 (5)	C52B—H52B	0.9300
C13A—H13A	0.9300	C52A—C53A	1.381 (5)
C14B—C15B	1.363 (5)	C52A—H52A	0.9300
C14B—Cl1B	1.735 (3)	C53B—C54B	1.370 (5)
C14A—C15A	1.363 (5)	C53B—H53B	0.9300
C14A—Cl1A	1.740 (3)	C53A—C54A	1.360 (6)
C15B—C16B	1.389 (5)	C53A—H53A	0.9300
C15B—H15B	0.9300	C54B—O3B	1.364 (4)
C15A—C16A	1.381 (4)	C54B—C55B	1.376 (4)
C15A—H15A	0.9300	C54A—O3A	1.357 (4)
C16B—H16B	0.9300	C54A—C55A	1.377 (5)
C16A—H16A	0.9300	C55B—C56B	1.380 (4)
C21B—O1B	1.207 (3)	C55B—H55B	0.9300
C21B—C22B	1.476 (4)	C55A—C56A	1.373 (5)
C21A—O1A	1.203 (3)	C55A—H55A	0.9300
C21A—C22A	1.492 (4)	C56B—H56B	0.9300
C22B—C23B	1.389 (4)	C56A—H56A	0.9300
C22B—C26B	1.392 (4)	N3B—H3B	0.90 (4)
C22A—C23A	1.384 (4)	N3A—H3A	0.84 (4)
C22A—C26A	1.387 (4)	O3B—C57B	1.405 (4)
C23B—C24B	1.363 (4)	O3A—C57A	1.411 (5)
C23B—H23B	0.9300	C57B—H57A	0.9600
C23A—C24A	1.363 (4)	C57B—H57C	0.9600
C23A—H23A	0.9300	C57B—H57B	0.9600
C24B—C25B	1.384 (4)	C57A—H57D	0.9600
C24B—H24B	0.9300	C57A—H57E	0.9600
C24A—C25A	1.382 (5)	C57A—H57F	0.9600
			
C11B—C1B—C4B	116.3 (2)	N2B—C26B—C22B	122.9 (3)
C11B—C1B—C2B	115.1 (2)	N2B—C26B—C27B	116.7 (3)
C4B—C1B—C2B	105.5 (2)	C22B—C26B—C27B	120.4 (3)
C11B—C1B—H1B	106.4	N2A—C26A—C22A	122.7 (3)
C4B—C1B—H1B	106.4	N2A—C26A—C27A	117.1 (3)
C2B—C1B—H1B	106.4	C22A—C26A—C27A	120.2 (3)
C11A—C1A—C4A	114.8 (2)	C26B—C27B—C28B	112.6 (2)
C11A—C1A—C2A	116.2 (2)	C26B—C27B—H27A	109.1
C4A—C1A—C2A	104.9 (2)	C28B—C27B—H27A	109.1
C11A—C1A—H1A	106.8	C26B—C27B—H27B	109.1
C4A—C1A—H1A	106.8	C28B—C27B—H27B	109.1
C2A—C1A—H1A	106.8	H27A—C27B—H27B	107.8
C28B—C2B—C21B	106.4 (2)	C26A—C27A—C28A	113.2 (2)
C28B—C2B—C1B	115.1 (2)	C26A—C27A—H27C	108.9
C21B—C2B—C1B	109.1 (2)	C28A—C27A—H27C	108.9
C28B—C2B—C3B	113.6 (2)	C26A—C27A—H27D	108.9
C21B—C2B—C3B	109.0 (2)	C28A—C27A—H27D	108.9
C1B—C2B—C3B	103.5 (2)	H27C—C27A—H27D	107.7
C28A—C2A—C21A	107.1 (2)	C27B—C28B—C2B	113.2 (2)
C28A—C2A—C1A	114.8 (2)	C27B—C28B—H28A	108.9
C21A—C2A—C1A	109.8 (2)	C2B—C28B—H28A	108.9
C28A—C2A—C3A	114.6 (2)	C27B—C28B—H28B	108.9
C21A—C2A—C3A	107.3 (2)	C2B—C28B—H28B	108.9
C1A—C2A—C3A	103.0 (2)	H28A—C28B—H28B	107.7
N1B—C3B—C31B	110.7 (2)	C2A—C28A—C27A	115.4 (2)
N1B—C3B—C37B	112.4 (2)	C2A—C28A—H28C	108.4
C31B—C3B—C37B	101.7 (2)	C27A—C28A—H28C	108.4
N1B—C3B—C2B	100.6 (2)	C2A—C28A—H28D	108.4
C31B—C3B—C2B	121.1 (2)	C27A—C28A—H28D	108.4
C37B—C3B—C2B	110.8 (2)	H28C—C28A—H28D	107.5
N1A—C3A—C31A	112.7 (2)	C36B—C31B—C32B	119.6 (3)
N1A—C3A—C37A	113.2 (2)	C36B—C31B—C3B	108.8 (3)
C31A—C3A—C37A	101.4 (2)	C32B—C31B—C3B	131.1 (3)
N1A—C3A—C2A	101.2 (2)	C32A—C31A—C36A	120.2 (3)
C31A—C3A—C2A	117.9 (2)	C32A—C31A—C3A	131.1 (3)
C37A—C3A—C2A	110.9 (2)	C36A—C31A—C3A	108.6 (3)
N1B—C4B—C5B	104.0 (2)	C31B—C32B—C33B	118.8 (3)
N1B—C4B—C1B	101.4 (2)	C31B—C32B—H32B	120.6
C5B—C4B—C1B	119.6 (3)	C33B—C32B—H32B	120.6
N1B—C4B—H4B	110.3	C31A—C32A—C33A	118.6 (3)
C5B—C4B—H4B	110.3	C31A—C32A—H32A	120.7
C1B—C4B—H4B	110.3	C33A—C32A—H32A	120.7
N1A—C4A—C5A	105.0 (2)	C34B—C33B—C32B	120.3 (3)
N1A—C4A—C1A	101.1 (2)	C34B—C33B—H33B	119.9
C5A—C4A—C1A	119.9 (3)	C32B—C33B—H33B	119.9
N1A—C4A—H4A	110.0	C34A—C33A—C32A	120.3 (4)
C5A—C4A—H4A	110.0	C34A—C33A—H33A	119.9
C1A—C4A—H4A	110.0	C32A—C33A—H33A	119.9
C4B—C5B—S1B	104.5 (2)	C35B—C34B—C33B	121.8 (3)
C4B—C5B—H5A	110.9	C35B—C34B—H34B	119.1
S1B—C5B—H5A	110.9	C33B—C34B—H34B	119.1
C4B—C5B—H5B	110.9	C33A—C34A—C35A	121.5 (3)
S1B—C5B—H5B	110.9	C33A—C34A—H34A	119.2
H5A—C5B—H5B	108.9	C35A—C34A—H34A	119.2
C4A—C5A—S1A	104.8 (2)	C34B—C35B—C36B	116.9 (3)
C4A—C5A—H5A1	110.8	C34B—C35B—H35B	121.6
S1A—C5A—H5A1	110.8	C36B—C35B—H35B	121.6
C4A—C5A—H5A2	110.8	C34A—C35A—C36A	118.0 (3)
S1A—C5A—H5A2	110.8	C34A—C35A—H35A	121.0
H5A1—C5A—H5A2	108.9	C36A—C35A—H35A	121.0
N1B—C6B—S1B	103.7 (2)	C31B—C36B—C35B	122.6 (3)
N1B—C6B—H6A	111.0	C31B—C36B—N3B	110.6 (3)
S1B—C6B—H6A	111.0	C35B—C36B—N3B	126.7 (3)
N1B—C6B—H6B	111.0	C35A—C36A—C31A	121.3 (3)
S1B—C6B—H6B	111.0	C35A—C36A—N3A	128.4 (3)
H6A—C6B—H6B	109.0	C31A—C36A—N3A	110.3 (3)
N1A—C6A—S1A	103.5 (2)	O2B—C37B—N3B	125.4 (3)
N1A—C6A—H6A1	111.1	O2B—C37B—C3B	126.4 (3)
S1A—C6A—H6A1	111.1	N3B—C37B—C3B	108.2 (2)
N1A—C6A—H6A2	111.1	O2A—C37A—N3A	125.3 (3)
S1A—C6A—H6A2	111.1	O2A—C37A—C3A	126.8 (3)
H6A1—C6A—H6A2	109.0	N3A—C37A—C3A	107.9 (3)
C16B—C11B—C12B	117.4 (3)	C56B—C51B—C52B	117.6 (3)
C16B—C11B—C1B	119.7 (3)	C56B—C51B—C25B	119.8 (3)
C12B—C11B—C1B	122.9 (3)	C52B—C51B—C25B	122.6 (3)
C12A—C11A—C16A	117.5 (3)	C56A—C51A—C52A	116.8 (3)
C12A—C11A—C1A	122.9 (3)	C56A—C51A—C25A	122.2 (3)
C16A—C11A—C1A	119.6 (3)	C52A—C51A—C25A	121.0 (3)
C11B—C12B—C13B	121.3 (3)	C53B—C52B—C51B	121.1 (3)
C11B—C12B—H12B	119.4	C53B—C52B—H52B	119.4
C13B—C12B—H12B	119.4	C51B—C52B—H52B	119.4
C11A—C12A—C13A	121.3 (3)	C51A—C52A—C53A	122.3 (4)
C11A—C12A—H12A	119.4	C51A—C52A—H52A	118.9
C13A—C12A—H12A	119.4	C53A—C52A—H52A	118.9
C14B—C13B—C12B	119.7 (3)	C52B—C53B—C54B	120.1 (3)
C14B—C13B—H13B	120.1	C52B—C53B—H53B	119.9
C12B—C13B—H13B	120.1	C54B—C53B—H53B	119.9
C14A—C13A—C12A	119.3 (3)	C54A—C53A—C52A	119.8 (4)
C14A—C13A—H13A	120.3	C54A—C53A—H53A	120.1
C12A—C13A—H13A	120.3	C52A—C53A—H53A	120.1
C13B—C14B—C15B	120.8 (3)	O3B—C54B—C53B	115.8 (3)
C13B—C14B—Cl1B	118.9 (3)	O3B—C54B—C55B	124.0 (3)
C15B—C14B—Cl1B	120.3 (3)	C53B—C54B—C55B	120.2 (3)
C13A—C14A—C15A	121.3 (3)	O3A—C54A—C53A	124.5 (4)
C13A—C14A—Cl1A	118.8 (3)	O3A—C54A—C55A	116.6 (4)
C15A—C14A—Cl1A	119.9 (3)	C53A—C54A—C55A	118.9 (3)
C14B—C15B—C16B	119.4 (3)	C54B—C55B—C56B	119.0 (3)
C14B—C15B—H15B	120.3	C54B—C55B—H55B	120.5
C16B—C15B—H15B	120.3	C56B—C55B—H55B	120.5
C14A—C15A—C16A	119.2 (3)	C56A—C55A—C54A	120.8 (4)
C14A—C15A—H15A	120.4	C56A—C55A—H55A	119.6
C16A—C15A—H15A	120.4	C54A—C55A—H55A	119.6
C11B—C16B—C15B	121.4 (3)	C51B—C56B—C55B	121.9 (3)
C11B—C16B—H16B	119.3	C51B—C56B—H56B	119.1
C15B—C16B—H16B	119.3	C55B—C56B—H56B	119.1
C15A—C16A—C11A	121.4 (3)	C55A—C56A—C51A	121.4 (3)
C15A—C16A—H16A	119.3	C55A—C56A—H56A	119.3
C11A—C16A—H16A	119.3	C51A—C56A—H56A	119.3
O1B—C21B—C22B	120.4 (3)	C6B—N1B—C3B	120.8 (2)
O1B—C21B—C2B	122.3 (3)	C6B—N1B—C4B	108.9 (2)
C22B—C21B—C2B	117.4 (2)	C3B—N1B—C4B	108.6 (2)
O1A—C21A—C22A	119.3 (3)	C6A—N1A—C4A	110.1 (2)
O1A—C21A—C2A	122.0 (3)	C6A—N1A—C3A	120.4 (2)
C22A—C21A—C2A	118.7 (2)	C4A—N1A—C3A	107.3 (2)
C23B—C22B—C26B	117.6 (3)	C26B—N2B—C25B	119.1 (3)
C23B—C22B—C21B	119.9 (3)	C25A—N2A—C26A	117.8 (3)
C26B—C22B—C21B	122.3 (3)	C37B—N3B—C36B	110.7 (3)
C23A—C22A—C26A	118.1 (3)	C37B—N3B—H3B	123 (2)
C23A—C22A—C21A	119.4 (3)	C36B—N3B—H3B	127 (2)
C26A—C22A—C21A	122.5 (3)	C37A—N3A—C36A	111.4 (3)
C24B—C23B—C22B	119.5 (3)	C37A—N3A—H3A	119 (3)
C24B—C23B—H23B	120.3	C36A—N3A—H3A	129 (3)
C22B—C23B—H23B	120.3	C54B—O3B—C57B	117.7 (3)
C24A—C23A—C22A	119.8 (3)	C54A—O3A—C57A	118.2 (4)
C24A—C23A—H23A	120.1	C6B—S1B—C5B	93.18 (14)
C22A—C23A—H23A	120.1	C6A—S1A—C5A	93.69 (14)
C23B—C24B—C25B	120.0 (3)	O3B—C57B—H57A	109.5
C23B—C24B—H24B	120.0	O3B—C57B—H57C	109.5
C25B—C24B—H24B	120.0	H57A—C57B—H57C	109.5
C23A—C24A—C25A	119.0 (3)	O3B—C57B—H57B	109.5
C23A—C24A—H24A	120.5	H57A—C57B—H57B	109.5
C25A—C24A—H24A	120.5	H57C—C57B—H57B	109.5
N2B—C25B—C24B	120.8 (3)	O3A—C57A—H57D	109.5
N2B—C25B—C51B	116.3 (3)	O3A—C57A—H57E	109.5
C24B—C25B—C51B	122.8 (3)	H57D—C57A—H57E	109.5
N2A—C25A—C24A	122.6 (3)	O3A—C57A—H57F	109.5
N2A—C25A—C51A	117.7 (3)	H57D—C57A—H57F	109.5
C24A—C25A—C51A	119.7 (3)	H57E—C57A—H57F	109.5
			
C11B—C1B—C2B—C28B	9.1 (4)	C37B—C3B—C31B—C36B	1.6 (3)
C4B—C1B—C2B—C28B	−120.5 (3)	C2B—C3B—C31B—C36B	124.8 (3)
C11B—C1B—C2B—C21B	−110.4 (3)	N1B—C3B—C31B—C32B	54.8 (4)
C4B—C1B—C2B—C21B	120.1 (3)	C37B—C3B—C31B—C32B	174.4 (3)
C11B—C1B—C2B—C3B	133.7 (2)	C2B—C3B—C31B—C32B	−62.4 (4)
C4B—C1B—C2B—C3B	4.1 (3)	N1A—C3A—C31A—C32A	−51.8 (4)
C11A—C1A—C2A—C28A	−10.6 (4)	C37A—C3A—C31A—C32A	−173.2 (3)
C4A—C1A—C2A—C28A	117.3 (3)	C2A—C3A—C31A—C32A	65.6 (4)
C11A—C1A—C2A—C21A	110.1 (3)	N1A—C3A—C31A—C36A	126.0 (3)
C4A—C1A—C2A—C21A	−122.0 (2)	C37A—C3A—C31A—C36A	4.6 (3)
C11A—C1A—C2A—C3A	−135.8 (2)	C2A—C3A—C31A—C36A	−116.6 (3)
C4A—C1A—C2A—C3A	−7.9 (3)	C36B—C31B—C32B—C33B	−2.5 (5)
C28B—C2B—C3B—N1B	147.3 (2)	C3B—C31B—C32B—C33B	−174.7 (3)
C21B—C2B—C3B—N1B	−94.3 (2)	C36A—C31A—C32A—C33A	3.5 (5)
C1B—C2B—C3B—N1B	21.8 (3)	C3A—C31A—C32A—C33A	−178.9 (3)
C28B—C2B—C3B—C31B	−90.5 (3)	C31B—C32B—C33B—C34B	1.0 (6)
C21B—C2B—C3B—C31B	28.0 (3)	C31A—C32A—C33A—C34A	−0.7 (6)
C1B—C2B—C3B—C31B	144.0 (3)	C32B—C33B—C34B—C35B	0.8 (6)
C28B—C2B—C3B—C37B	28.3 (3)	C32A—C33A—C34A—C35A	−1.8 (7)
C21B—C2B—C3B—C37B	146.7 (2)	C33B—C34B—C35B—C36B	−1.0 (6)
C1B—C2B—C3B—C37B	−97.3 (3)	C33A—C34A—C35A—C36A	1.3 (6)
C28A—C2A—C3A—N1A	−145.2 (2)	C32B—C31B—C36B—C35B	2.3 (5)
C21A—C2A—C3A—N1A	96.1 (2)	C3B—C31B—C36B—C35B	176.1 (3)
C1A—C2A—C3A—N1A	−19.7 (3)	C32B—C31B—C36B—N3B	−175.2 (3)
C28A—C2A—C3A—C31A	91.5 (3)	C3B—C31B—C36B—N3B	−1.4 (4)
C21A—C2A—C3A—C31A	−27.2 (3)	C34B—C35B—C36B—C31B	−0.6 (5)
C1A—C2A—C3A—C31A	−143.1 (2)	C34B—C35B—C36B—N3B	176.6 (3)
C28A—C2A—C3A—C37A	−24.7 (3)	C34A—C35A—C36A—C31A	1.6 (6)
C21A—C2A—C3A—C37A	−143.5 (2)	C34A—C35A—C36A—N3A	−177.2 (4)
C1A—C2A—C3A—C37A	100.7 (2)	C32A—C31A—C36A—C35A	−4.0 (5)
C11B—C1B—C4B—N1B	−157.4 (2)	C3A—C31A—C36A—C35A	177.9 (3)
C2B—C1B—C4B—N1B	−28.5 (3)	C32A—C31A—C36A—N3A	175.0 (3)
C11B—C1B—C4B—C5B	89.0 (3)	C3A—C31A—C36A—N3A	−3.1 (4)
C2B—C1B—C4B—C5B	−142.1 (3)	N1B—C3B—C37B—O2B	−60.6 (4)
C11A—C1A—C4A—N1A	161.6 (2)	C31B—C3B—C37B—O2B	−179.1 (3)
C2A—C1A—C4A—N1A	32.8 (3)	C2B—C3B—C37B—O2B	51.0 (4)
C11A—C1A—C4A—C5A	−83.7 (3)	N1B—C3B—C37B—N3B	117.2 (3)
C2A—C1A—C4A—C5A	147.5 (3)	C31B—C3B—C37B—N3B	−1.2 (3)
N1B—C4B—C5B—S1B	38.7 (3)	C2B—C3B—C37B—N3B	−131.2 (3)
C1B—C4B—C5B—S1B	150.9 (2)	N1A—C3A—C37A—O2A	52.9 (4)
N1A—C4A—C5A—S1A	−35.1 (3)	C31A—C3A—C37A—O2A	173.9 (3)
C1A—C4A—C5A—S1A	−147.7 (2)	C2A—C3A—C37A—O2A	−60.0 (4)
C4B—C1B—C11B—C16B	−142.6 (3)	N1A—C3A—C37A—N3A	−125.8 (3)
C2B—C1B—C11B—C16B	93.4 (3)	C31A—C3A—C37A—N3A	−4.7 (3)
C4B—C1B—C11B—C12B	37.7 (4)	C2A—C3A—C37A—N3A	121.3 (3)
C2B—C1B—C11B—C12B	−86.3 (4)	N2B—C25B—C51B—C56B	−6.5 (5)
C4A—C1A—C11A—C12A	−49.6 (4)	C24B—C25B—C51B—C56B	173.3 (3)
C2A—C1A—C11A—C12A	73.3 (4)	N2B—C25B—C51B—C52B	173.2 (3)
C4A—C1A—C11A—C16A	129.4 (3)	C24B—C25B—C51B—C52B	−7.0 (5)
C2A—C1A—C11A—C16A	−107.7 (3)	N2A—C25A—C51A—C56A	37.5 (5)
C16B—C11B—C12B—C13B	−0.6 (5)	C24A—C25A—C51A—C56A	−142.6 (4)
C1B—C11B—C12B—C13B	179.0 (3)	N2A—C25A—C51A—C52A	−143.9 (4)
C16A—C11A—C12A—C13A	0.7 (5)	C24A—C25A—C51A—C52A	36.0 (5)
C1A—C11A—C12A—C13A	179.7 (3)	C56B—C51B—C52B—C53B	−0.3 (5)
C11B—C12B—C13B—C14B	−0.7 (5)	C25B—C51B—C52B—C53B	180.0 (3)
C11A—C12A—C13A—C14A	−0.2 (5)	C56A—C51A—C52A—C53A	−0.9 (6)
C12B—C13B—C14B—C15B	1.5 (5)	C25A—C51A—C52A—C53A	−179.6 (4)
C12B—C13B—C14B—Cl1B	−179.2 (3)	C51B—C52B—C53B—C54B	0.9 (6)
C12A—C13A—C14A—C15A	0.3 (5)	C51A—C52A—C53A—C54A	−0.1 (7)
C12A—C13A—C14A—Cl1A	179.1 (2)	C52B—C53B—C54B—O3B	179.5 (3)
C13B—C14B—C15B—C16B	−0.8 (5)	C52B—C53B—C54B—C55B	−1.5 (5)
Cl1B—C14B—C15B—C16B	179.8 (3)	C52A—C53A—C54A—O3A	179.5 (4)
C13A—C14A—C15A—C16A	−0.9 (5)	C52A—C53A—C54A—C55A	1.4 (7)
Cl1A—C14A—C15A—C16A	−179.7 (2)	O3B—C54B—C55B—C56B	−179.6 (3)
C12B—C11B—C16B—C15B	1.3 (5)	C53B—C54B—C55B—C56B	1.5 (5)
C1B—C11B—C16B—C15B	−178.4 (3)	O3A—C54A—C55A—C56A	179.9 (4)
C14B—C15B—C16B—C11B	−0.6 (5)	C53A—C54A—C55A—C56A	−1.8 (6)
C14A—C15A—C16A—C11A	1.4 (5)	C52B—C51B—C56B—C55B	0.3 (5)
C12A—C11A—C16A—C15A	−1.3 (4)	C25B—C51B—C56B—C55B	−180.0 (3)
C1A—C11A—C16A—C15A	179.6 (3)	C54B—C55B—C56B—C51B	−0.9 (5)
C28B—C2B—C21B—O1B	−141.2 (3)	C54A—C55A—C56A—C51A	0.8 (6)
C1B—C2B—C21B—O1B	−16.5 (4)	C52A—C51A—C56A—C55A	0.5 (6)
C3B—C2B—C21B—O1B	95.9 (3)	C25A—C51A—C56A—C55A	179.2 (3)
C28B—C2B—C21B—C22B	38.4 (3)	S1B—C6B—N1B—C3B	170.4 (2)
C1B—C2B—C21B—C22B	163.1 (2)	S1B—C6B—N1B—C4B	43.7 (3)
C3B—C2B—C21B—C22B	−84.5 (3)	C31B—C3B—N1B—C6B	60.9 (3)
C28A—C2A—C21A—O1A	147.6 (3)	C37B—C3B—N1B—C6B	−52.0 (3)
C1A—C2A—C21A—O1A	22.3 (4)	C2B—C3B—N1B—C6B	−169.9 (2)
C3A—C2A—C21A—O1A	−89.0 (3)	C31B—C3B—N1B—C4B	−172.2 (2)
C28A—C2A—C21A—C22A	−35.4 (3)	C37B—C3B—N1B—C4B	74.9 (3)
C1A—C2A—C21A—C22A	−160.7 (2)	C2B—C3B—N1B—C4B	−43.0 (3)
C3A—C2A—C21A—C22A	88.0 (3)	C5B—C4B—N1B—C6B	−55.4 (3)
O1B—C21B—C22B—C23B	−11.0 (5)	C1B—C4B—N1B—C6B	179.8 (2)
C2B—C21B—C22B—C23B	169.4 (3)	C5B—C4B—N1B—C3B	171.2 (2)
O1B—C21B—C22B—C26B	173.7 (3)	C1B—C4B—N1B—C3B	46.4 (3)
C2B—C21B—C22B—C26B	−6.0 (4)	S1A—C6A—N1A—C4A	−43.0 (3)
O1A—C21A—C22A—C23A	8.0 (5)	S1A—C6A—N1A—C3A	−168.6 (2)
C2A—C21A—C22A—C23A	−169.1 (3)	C5A—C4A—N1A—C6A	52.7 (3)
O1A—C21A—C22A—C26A	−172.8 (3)	C1A—C4A—N1A—C6A	178.1 (2)
C2A—C21A—C22A—C26A	10.2 (4)	C5A—C4A—N1A—C3A	−174.6 (2)
C26B—C22B—C23B—C24B	2.1 (5)	C1A—C4A—N1A—C3A	−49.2 (3)
C21B—C22B—C23B—C24B	−173.5 (3)	C31A—C3A—N1A—C6A	−63.0 (3)
C26A—C22A—C23A—C24A	−1.0 (5)	C37A—C3A—N1A—C6A	51.4 (4)
C21A—C22A—C23A—C24A	178.2 (3)	C2A—C3A—N1A—C6A	170.1 (3)
C22B—C23B—C24B—C25B	1.1 (5)	C31A—C3A—N1A—C4A	170.1 (2)
C22A—C23A—C24A—C25A	0.9 (5)	C37A—C3A—N1A—C4A	−75.5 (3)
C23B—C24B—C25B—N2B	−3.3 (5)	C2A—C3A—N1A—C4A	43.3 (3)
C23B—C24B—C25B—C51B	176.9 (3)	C22B—C26B—N2B—C25B	1.2 (5)
C23A—C24A—C25A—N2A	−0.2 (5)	C27B—C26B—N2B—C25B	−178.7 (3)
C23A—C24A—C25A—C51A	179.9 (3)	C24B—C25B—N2B—C26B	2.2 (5)
C23B—C22B—C26B—N2B	−3.3 (5)	C51B—C25B—N2B—C26B	−178.0 (3)
C21B—C22B—C26B—N2B	172.1 (3)	C24A—C25A—N2A—C26A	−0.4 (5)
C23B—C22B—C26B—C27B	176.5 (3)	C51A—C25A—N2A—C26A	179.5 (3)
C21B—C22B—C26B—C27B	−8.0 (5)	C22A—C26A—N2A—C25A	0.2 (4)
C23A—C22A—C26A—N2A	0.5 (4)	C27A—C26A—N2A—C25A	−178.0 (3)
C21A—C22A—C26A—N2A	−178.8 (3)	O2B—C37B—N3B—C36B	178.3 (3)
C23A—C22A—C26A—C27A	178.6 (3)	C3B—C37B—N3B—C36B	0.5 (3)
C21A—C22A—C26A—C27A	−0.6 (4)	C31B—C36B—N3B—C37B	0.6 (4)
N2B—C26B—C27B—C28B	166.4 (3)	C35B—C36B—N3B—C37B	−176.8 (3)
C22B—C26B—C27B—C28B	−13.5 (4)	O2A—C37A—N3A—C36A	−175.5 (3)
N2A—C26A—C27A—C28A	−163.0 (3)	C3A—C37A—N3A—C36A	3.2 (3)
C22A—C26A—C27A—C28A	18.8 (4)	C35A—C36A—N3A—C37A	178.8 (4)
C26B—C27B—C28B—C2B	49.4 (4)	C31A—C36A—N3A—C37A	−0.2 (4)
C21B—C2B—C28B—C27B	−60.4 (3)	C53B—C54B—O3B—C57B	−175.7 (3)
C1B—C2B—C28B—C27B	178.6 (2)	C55B—C54B—O3B—C57B	5.3 (5)
C3B—C2B—C28B—C27B	59.5 (3)	C53A—C54A—O3A—C57A	−1.5 (7)
C21A—C2A—C28A—C27A	54.7 (3)	C55A—C54A—O3A—C57A	176.7 (4)
C1A—C2A—C28A—C27A	176.9 (2)	N1B—C6B—S1B—C5B	−16.2 (2)
C3A—C2A—C28A—C27A	−64.2 (3)	C4B—C5B—S1B—C6B	−13.1 (2)
C26A—C27A—C28A—C2A	−48.1 (4)	N1A—C6A—S1A—C5A	17.3 (2)
N1B—C3B—C31B—C36B	−118.0 (3)	C4A—C5A—S1A—C6A	10.3 (2)

### Hydrogen-bond geometry (Å, º)

*Cg*8 and *Cg*17 are the centroids of the C51*A*–C56*A* and C11*B*–C16*B* rings, respectively.

**Table:**

*D*—H···*A*	*D*—H	H···*A*	*D*···*A*	*D*—H···*A*
N3*A*—H3*A*···O2*A*^i^	0.83 (4)	2.05 (4)	2.874 (4)	172 (4)
N3*B*—H3*B*···N2*A*^ii^	0.91 (4)	2.24 (4)	3.136 (4)	169 (3)
C23*B*—H23*B*···O1*A*	0.93	2.36	3.066 (4)	132
C27*A*—H27*D*···O2*B*^iii^	0.97	2.40	3.371 (4)	177
C28*A*—H28*C*···O3*B*^iv^	0.97	2.55	3.517 (4)	175
C57*B*—H57*C*···O2*B*^v^	0.96	2.50	3.349 (5)	147
C5*A*—H5*A*1···*Cg*17^vi^	0.97	2.91	3.711 (4)	140
C27*B*—H27*A*···*Cg*8^iv^	0.97	2.89	3.783 (4)	154

Symmetry codes: (i) −*x*, −*y*+1, −*z*+1; (ii) *x*, *y*−1, *z*; (iii) *x*, *y*+1, *z*; (iv) −*x*+1, −*y*+1, −*z*; (v) −*x*+1, −*y*, −*z*; (vi) −*x*+1, −*y*, −*z*+1.
