# Supplementary material for: Crystal structure of 7′-(4-chloro­phen­yl)-2′′-(4-meth­oxy­phen­yl)-7′,7a’,7′′,8′′-tetra­hydro-1′H,3′H,5′′H-di­spiro­[indoline-3,5′-pyrrolo­[1,2-c]thia­zole-6′,6′′-quinoline]-2,5′′-dione and an unknown solvent
Source: Acta Crystallogr E Crystallogr Commun. 2019 Jan 11;75(Pt 2):189–93. doi: 10.1107/S2056989019000112 (PMC6362668; doi:10.1107/S2056989019000112)
Supplement: Supplementary file 3 [file e-75-00189-sup3.pdf]

Search Overview

Search:	search2
Date/Time done:	Tue Jan 01 16:11:28 2019
Database(s):	CSD version 5.39 updates (Nov 2017)<br>CSD version 5.39 (November 2017)<br>CSD version 5.39 (November 2017)<br>CSD version 5.39 updates (Feb 2018)<br>CSD version 5.39 updates (May 2018)<br>CSD version 5.39 updates (Aug 2018)
Restriction Info:	No refcode restrictions applied
Filters:	None
Percentage Completed:	100%
Number of Hits:	16

Single query used. Search found structures that:

match

Query 1

Query 1

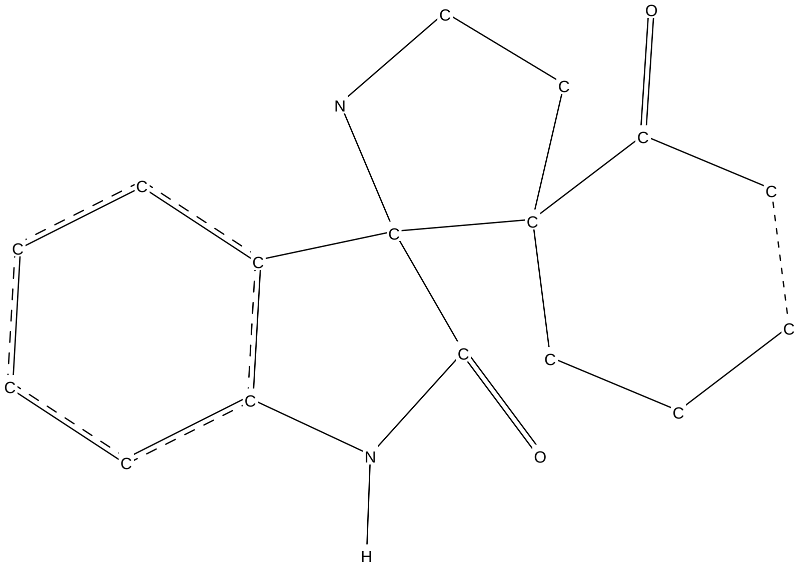

Search: search2 (Tue Jan 01 16:11:28 2019): Hit 1

WEFKEA

Reference:	Demin Ren, Guoqiang Kuang, Xiaolian Hu, Xiaofang Li<br>(2017) *J.Chem.Res.* ,**41,**427
Formula:	$C_{33} H_{32} N_4 O_5$
Compound Name:	1'-methyl-1-phenyl-4'-(3,4,5-trimethoxyphenyl)-6,7-dihydrodispiro[indazole-5,3'-pyrrolidine-2',3''-indole]-2'',4(1H,1''H)-dione

Space Group:	P21/c	***Cell:***	***a*** 12.141(1)	***b*** 13.563(1)	***c*** 17.660(2)
Space Group No.:	14	***(Å,°)***	α 90.00	β 103.87(0)	γ 90.00

R-Factor (%):	4.54	***Temperature(K):***	113	***Density(g/cm³):***	1.328

Parameters

Fragment 1

**ANG1 (A)**	83.365
**ANG2 (A)**	86.391
**ANG3 (A)**	47.257

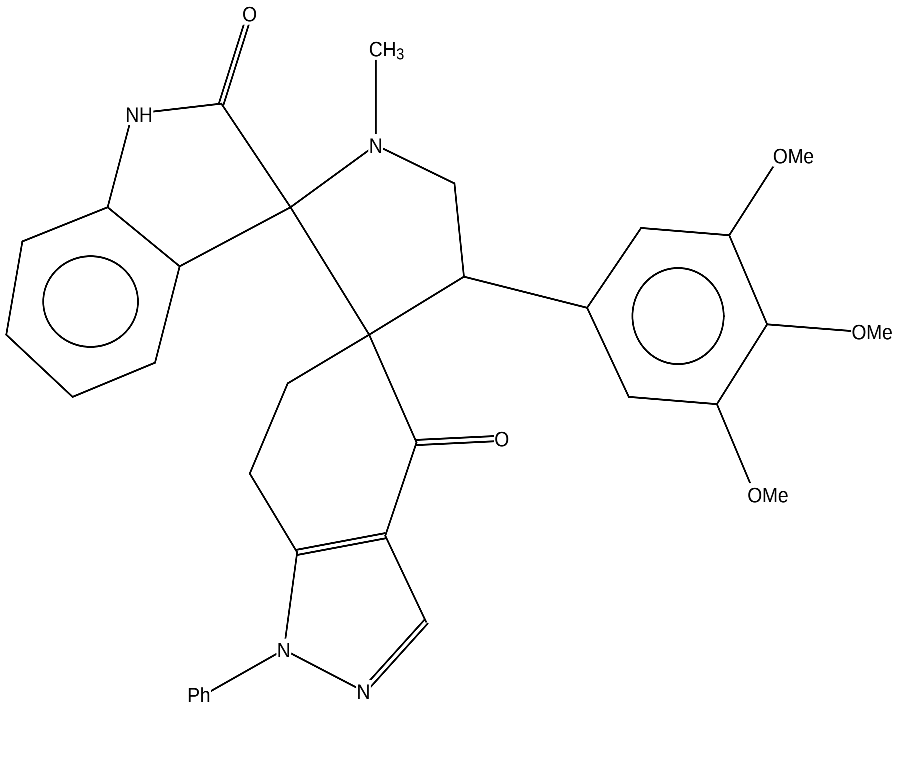

Search: search2 (Tue Jan 01 16:11:28 2019): Hit 2

BOQXOU

Reference:	A.Jeyabharathi, M.N.Ponnuswamy, A.A.Raj, R.Raghunathan, I.A.Razak, A.Usman, S.Chantrapromma, H.-K.Fun (2001) *Acta Crystallogr.,Sect.E:Struct.Rep.Online* ,**57,**o901
Formula:	C_{30} H_{28} N_2 O_2
Compound Name:	3-Benzylidene-1'-methyl-4'-phenylcyclohexanespiro-3'-pyrrolidine-2'-spiro-3''-indoline-2,2''-dione

Space Group:	P-1	***Cell:***	***a*** 12.362(0)	***b*** 13.064(0)	***c*** 17.144(0)
Space Group No.:	2	***(Å,°)***	α 110.07(0)	β 93.20(0)	γ 110.53(0)
R-Factor (%):	5.70	***Temperature(K):***	293	***Density(g/cm³):***	1.249

Parameters

Fragment 1

**ANG1 (A)**	86.012
**ANG2 (A)**	85.281
**ANG3 (A)**	56.543

Fragment 2

**ANG1 (A)**	87.861
**ANG2 (A)**	84.281
**ANG3 (A)**	57.585

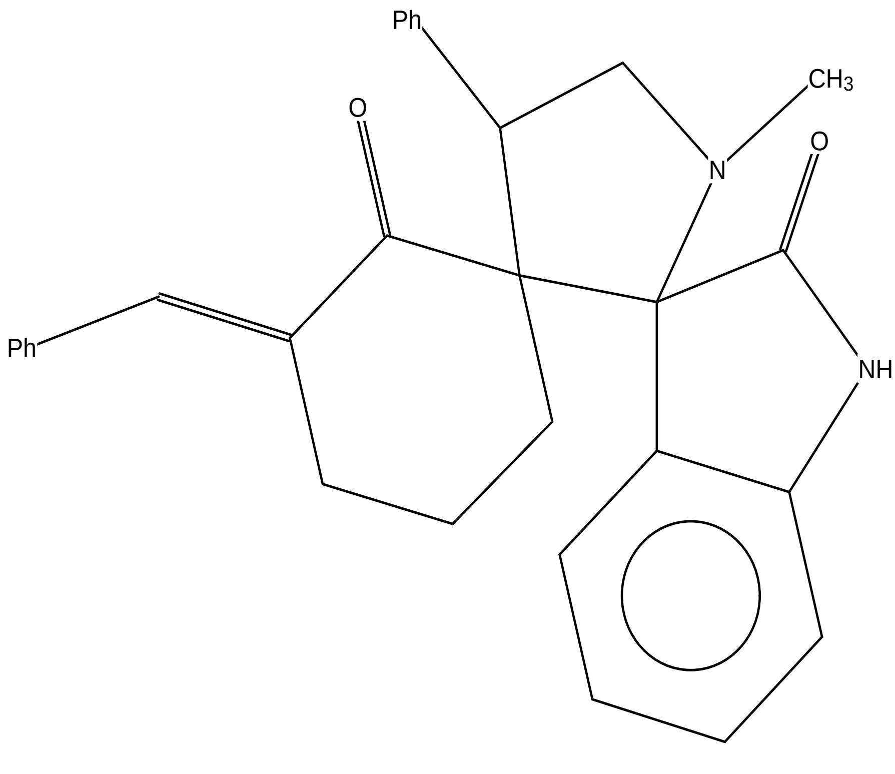

Search: search2 (Tue Jan 01 16:11:28 2019): Hit 3

EJAJEF

Reference:	N.Sampath, R.Mathews, M.N.Ponnuswamy (2010)<br>*J.Chem.Cryst.* ,**40,**1105
Formula:	$C_{30} H_{28} N_2 O_2,C_4 H_{10} O_1$
Compound Name:	1'-Phenyl-1',5',10',10a'-tetrahydro-2H-dispiro[cyclohexane-1,2'-pyrrolo[1,2-b]isoquinoline-3',3''-indole]-2,2''(1''H)-dione diethyl ether solvate

Space Group:	P21/a	***Cell:***	***a*** 11.959(6)	***b*** 19.308(3)	***c*** 13.452(6)
Space Group No.:	14	***(Å,°)***	α 90.00	β 107.81(4)	γ 90.00
R-Factor (%):	8.79	***Temperature(K):***	293	***Density(g/cm³):***	1.174

Parameters

Fragment 1

**ANG1 (A)**	89.005
**ANG2 (A)**	85.634
**ANG3 (A)**	50.447

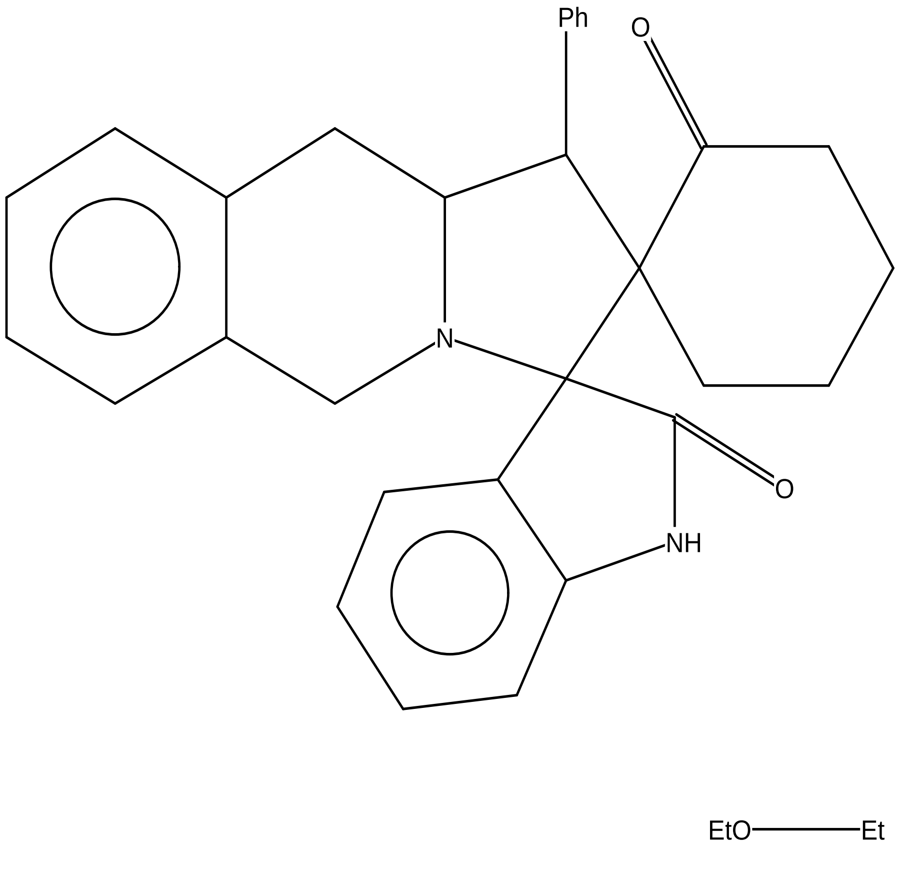

Search: search2 (Tue Jan 01 16:11:28 2019): Hit 4

LOBCIP

Reference:	G.Periyasami, R.Raghunathan, G.Surendiran, N.Mathivanan (2008) *Bioorg.Med.Chem.Lett.* ,**18,**2342
Formula:	$C_{32}\ H_{29}\ N_3\ O_2$
Compound Name:	1'-p-Tolyl-4,5',6',7',7a',9-hexahydro-1'H-dispiro[carbazole-2,2'-pyrrolizine-3',3''-indole]-1,2''(1''H,3H)-dione
Synonym:	2'-(3,4-tetrahydro-1-ketocarbazole)-(1'-(p-methyl)phenyl)-2',3',3a',4',5',6'-hexahydro(2.2',3.3')dispiropyrrolizidineoxindole

Space Group:	P21/c	***Cell:***	***a*** 16.469(1)	***b***	8.726(0)	***c***	20.502(1)
Space Group No.:	14	***(Å,°)***	α 90.00	β	100.16(0)	γ	90.00
R-Factor (%):	37.30	***Temperature(K):***	293	***Density(g/cm^3):***	1.117		

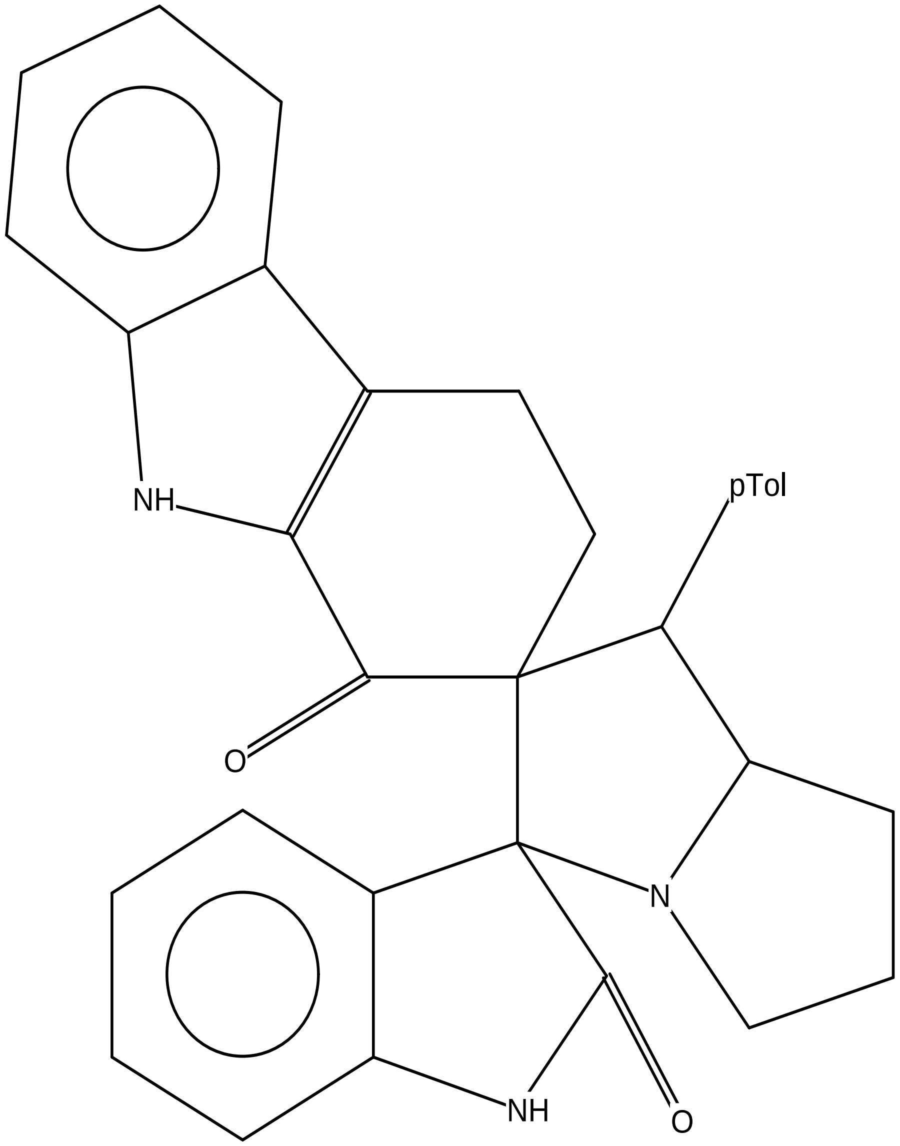

Parameters

Fragment 1

**ANG1 (A)**	80.604
**ANG2 (A)**	88.983
**ANG3 (A)**	52.486

Search: search2 (Tue Jan 01 16:11:28 2019): Hit 5

NAQCAL

Reference:	S.U.Maheswari, S.Perumal, A.I.Almansour (2012)<br>*Tetrahedron Lett.* ,**53,**349
Formula:	$C_{30}\ H_{24}\ Cl_1\ N_3\ O_2,C_1\ H_4\ O_1$
Compound Name:	4'-(4-Chlorophenyl)-1'-methyl-3,4-dihydro-1H-dispiro[acridine-2,3'-pyrrolidine-2',3''-indole]-1,2''(1''H)-dione methanol solvate

Space Group:	P-1	***Cell:***	***a*** 10.328(3)	***b*** 16.696(4)	***c*** 16.775(5)
Space Group No.:	2	***(Å,°)***	α 109.83(3)	β 101.66(2)	γ 95.85(2)
R-Factor (%):	4.63	***Temperature(K):*** 293		***Density(g/cm³):*** 1.334	

Parameters

Fragment 1

**ANG1 (A)**	83.302
**ANG2 (A)**	88.253
**ANG3 (A)**	45.584

Fragment 2

**ANG1 (A)**	86.341
**ANG2 (A)**	85.152
**ANG3 (A)**	52.781

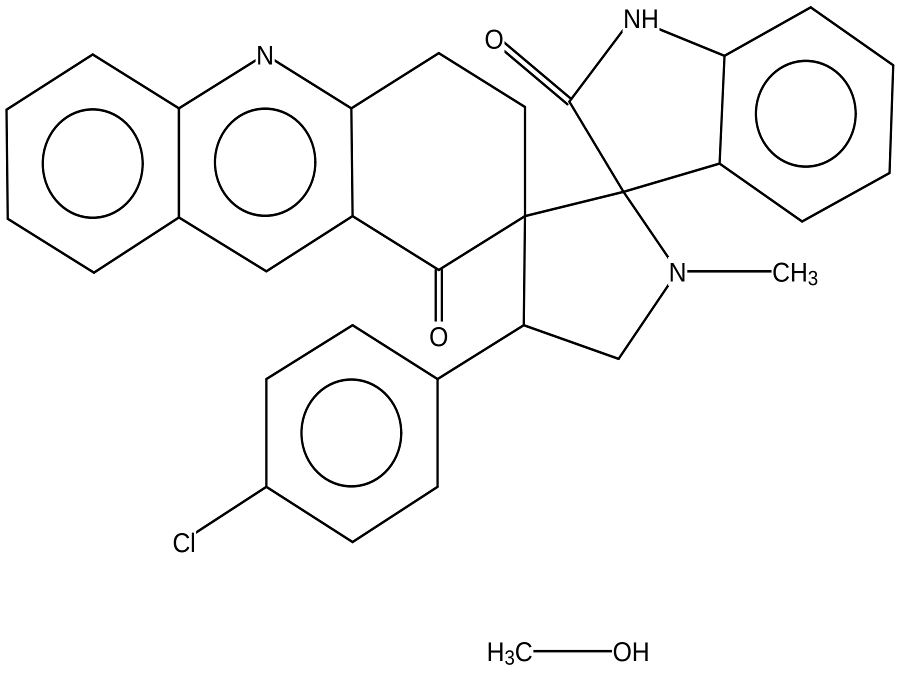

Search: search2 (Tue Jan 01 16:11:28 2019): Hit 6

RAGMUK

Reference:	G.Lotfy, M.M.Said, El S.H El Ashry, El S.H El Tamany, A.Al-Dhfyan, Y.M.A.Aziz, A.Barakat (2017) *Bioorg.Med.Chem.* ,**25,**1514
Formula:	C_{31} H_{28} N_2 O_2 S_1
Compound Name:	(1R,5'R)-3-benzylidene-7'-phenyl-7',7a'-dihydro-1'H,2H-dispiro[cyclohexane-1,6'-pyrrolo[1,2-c][1,3]thiazole-5',3''-indole]-2,2''(1''H)-dione

Space Group:	P-1	***Cell:***	***a*** 7.772(0)	***b*** 10.508(0)	***c*** 15.496(0)
Space Group No.:	2	***(Å,°)***	α 97.10(0)	β 100.91(0)	γ 97.84(0)

R-Factor (%):	5.59	***Temperature(K):***	100	***Density(g/cm^3):***	1.345

Parameters

Fragment 1

**ANG1 (A)**	83.636
**ANG2 (A)**	89.204
**ANG3 (A)**	52.247

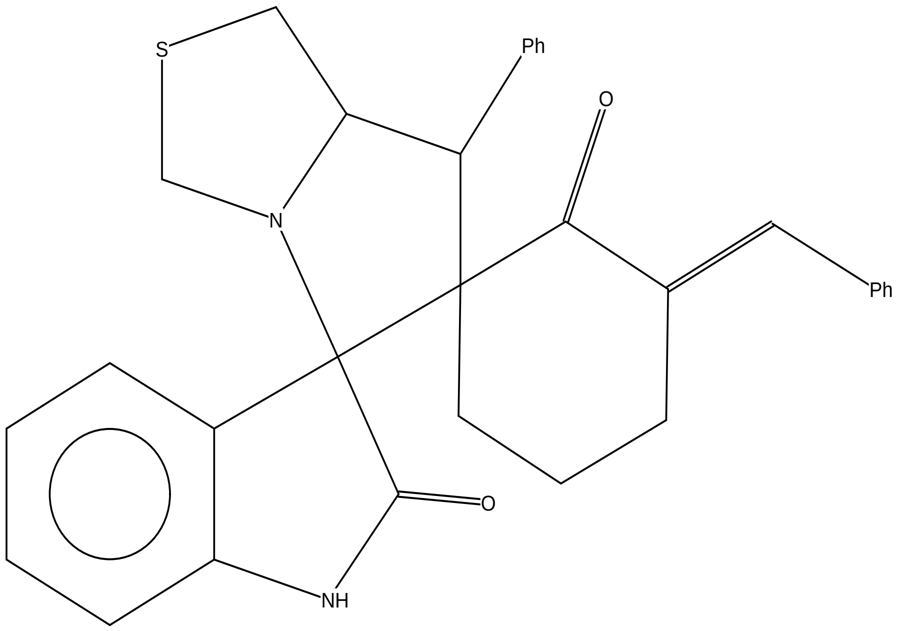

Search: search2 (Tue Jan 01 16:11:28 2019): Hit 7

RAGNAR

Reference: G.Lotfy, M.M.Said, El S.H El Ashry, El S.H El Tamany, A.Al-Dhfyan, Y.M.A.Aziz, A.Barakat (2017) *Bioorg.Med.Chem.* ,**25,**1514

Formula: $C_{33} H_{32} N_2 O_2 S_1$

Compound Name: (1R,5'R)-3-(3-methylbenzylidene)-7'-(3-methylphenyl)-7',7a'-dihydro-1'H, 2H-dispiro[cyclohexane-1,6'-pyrrolo[1,2-c][1,3]thiazole-5',3''-indole]-2, 2''(1''H)-dione

Space Group:	P-1	***Cell:***	***a***	7.662(1)	***b***	10.634(3)	***c***	16.593(4)
Space Group No.:	2	***(Å,°)***	α	93.93(0)	β	98.07(0)	γ	91.40(0)

R-Factor (%): 10.79 ***Temperature(K):*** 100 ***Density(g/cm^3):*** 1.296

Parameters

Fragment 1

**ANG1 (A)**	82.989
**ANG2 (A)**	88.596
**ANG3 (A)**	55.749

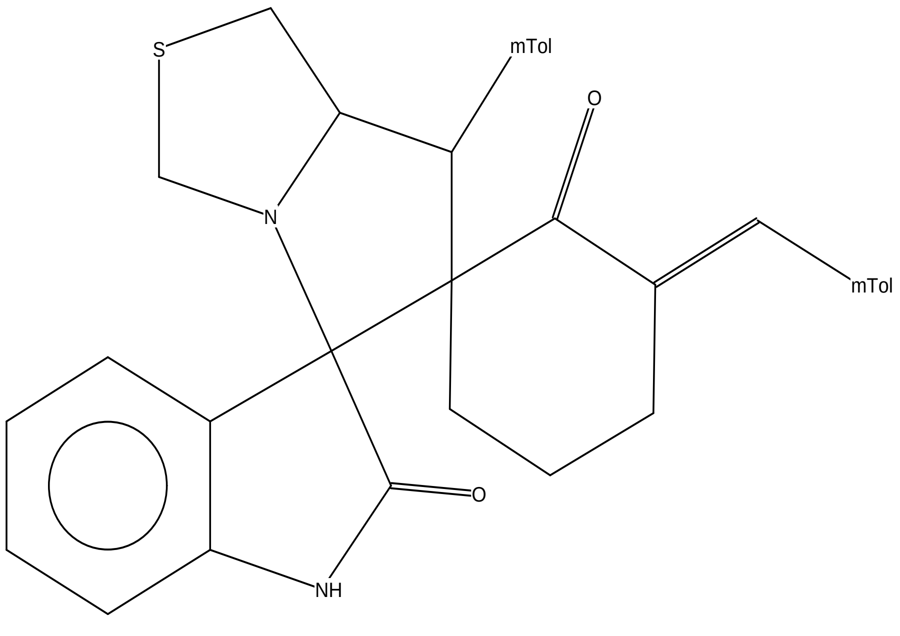

Search: search2 (Tue Jan 01 16:11:28 2019): Hit 8

RAHBIO

Reference: G.Lotfy, M.M.Said, El S.H El Ashry, El S.H El Tamany, A.Al-Dhfyan, Y.M.A.Aziz, A.Barakat (2017) *Bioorg.Med.Chem.* ,**25,**1514

Formula: $C_{27} H_{24} N_2 O_2 S_3$

Compound Name: (1R,5'R,E)-7'-(2-thienyl)-3-(2-thienylmethylene)-7',7a'-dihydro-1'H,2H-dispiro[cyclohexane-1,6'-pyrrolo[1,2-c][1,3]thiazole-5',3''-indole]-2,2''(1''H)-dione

Space Group:	P-1	***Cell:***	***a*** 7.828(1)	***b*** 10.445(1)	***c*** 15.031(2)		
Space Group No.:	2	***(Å,°)***	α 96.63(0)	β 100.78(0)	γ 99.81(0)		
R-Factor (%):	8.27	***Temperature(K):***	100	***Density(g/cm³):***	1.425		

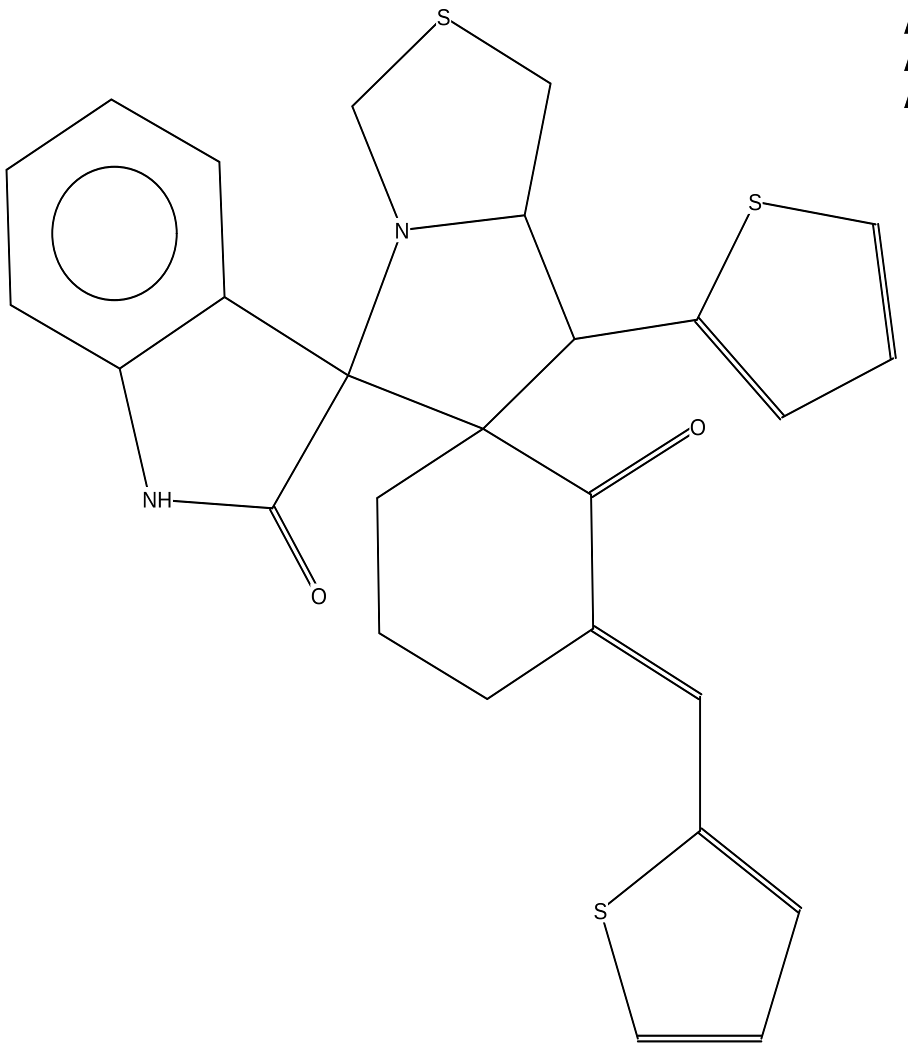

Parameters

Fragment 1

**ANG1 (A)**	83.594
**ANG2 (A)**	89.563
**ANG3 (A)**	49.278

Search: search2 (Tue Jan 01 16:11:28 2019): Hit 9

ROFQOT

Reference:	E.T.S.Kamala, R.Murugan, S.Nirmala, L.Sudha, S.S.Narayanan (2008) *Acta Crystallogr.,Sect.E:Struct.Rep.Online* ,**64,** o1958
Formula:	$C_{37} H_{32} Cl_2 N_2 O_4$
Compound Name:	(1'S)-4-(3,4-Dichlorophenyl)-1'-(3,5-dimethoxyphenyl)-1,2,3,4-tetrahydronaphthalene-2-spiro-2'-pyrrolizidine-3'-spiro-3''-indoline-1,2''-dione

Space Group:	P-1	***Cell:***	***a***	10.447(0)	***b***	11.305(0)	***c***	15.017(0)
Space Group No.:	2	***(Å,°)***	α	87.92(0)	β	70.32(0)	γ	70.11(0)

R-Factor (%):	5.63	***Temperature(K):***	293	***Density(g/cm^3):***	1.358

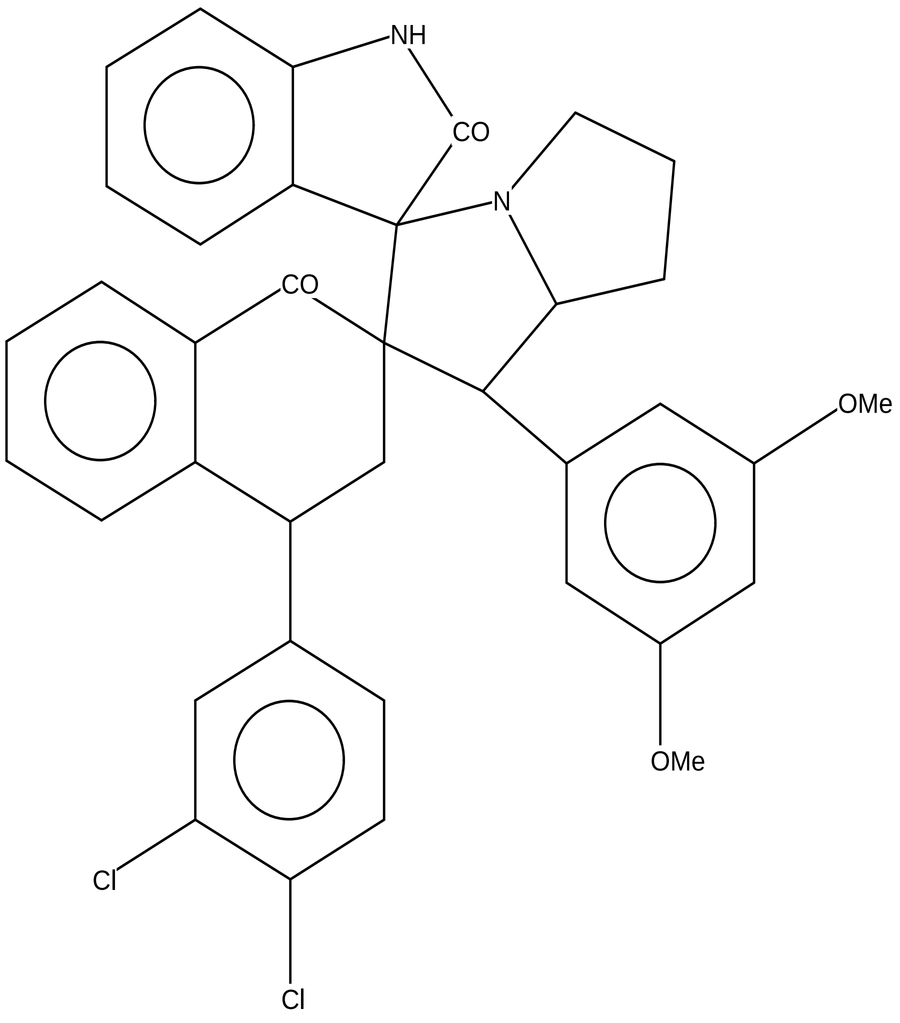

Parameters

Fragment 1

**ANG1 (A)**	80.054
**ANG2 (A)**	86.991
**ANG3 (A)**	49.657

Search: search2 (Tue Jan 01 16:11:28 2019): Hit 10

TEJRUW

Reference:	A.Subbiahpandi, D.Velmurugan, K.Ravikumar, E.Ramesh, R.Raghunathan (2006) *Acta Crystallogr.,Sect.E:Struct.Rep.Online* ,**62,** o2259
Formula:	$C_{37}\ H_{32}\ N_2\ O_2\ S_1$
Compound Name:	3-Benzylidene-3',7'-diphenylcyclohexanespiro-6'-(perhydro-2-thiapyrrolizine)-5'-spiro-3''-(1H-indole)-2,2''-dione

Space Group:	P21/c	***Cell:***	***a*** 10.031(0)	***b*** 20.860(1)	***c*** 14.131(0)
Space Group No.:	14	***(Å,°)***	α 90.00	β 100.97(0)	γ 90.00

R-Factor (%):	5.14	***Temperature(K):***	293	***Density(g/cm³):***	1.301

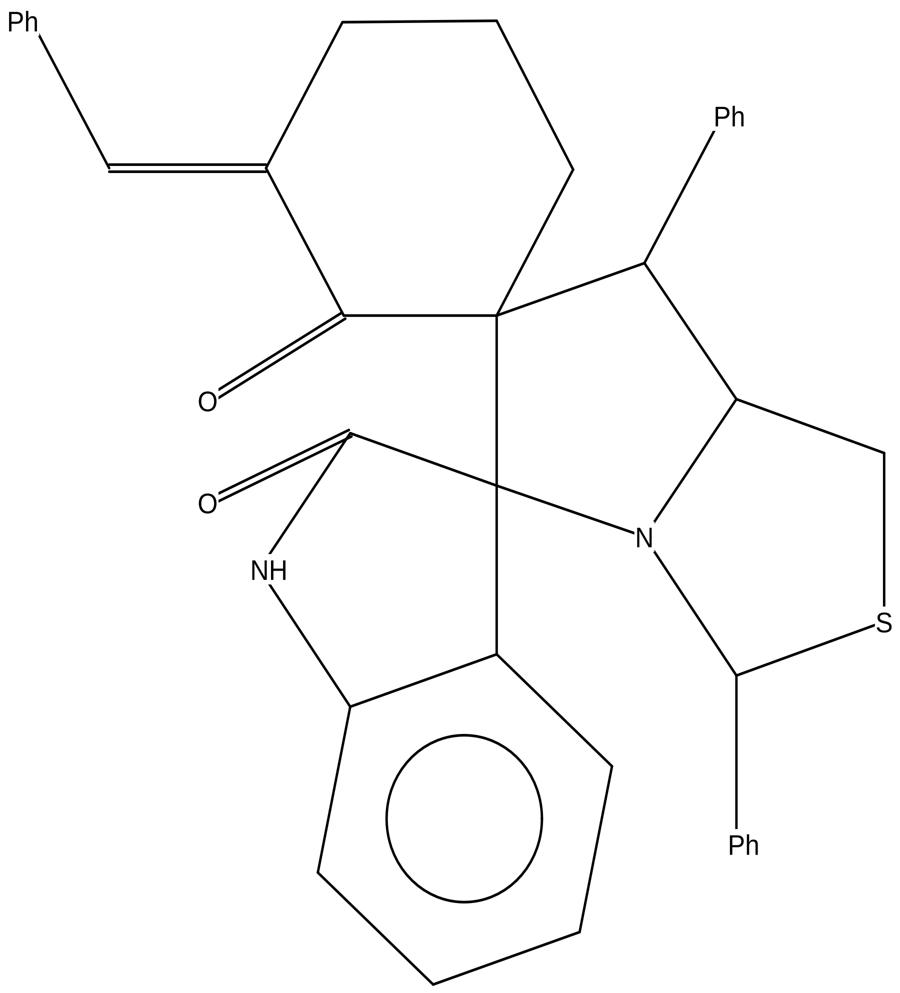

Parameters

Fragment 1

**ANG1 (A)**	81.015
**ANG2 (A)**	86.521
**ANG3 (A)**	50.995

Search: search2 (Tue Jan 01 16:11:28 2019): Hit 11

UQIROD

Reference: R.V.Sumesh, M.Muthu, A.I.Almansour, R.S.Kumar, N.Arumugam, S.Athimoolam, E.Arockia Jeya Yasmi Prabha, R.R.Kumar (2016) *ACS Comb. Sci.* ,**18,**262

Formula: $C_{34}\ H_{27}\ Cl_2\ N_5\ O_2$,x($C_1\ H_1\ Cl_3$)

Compound Name: 4'-(2,4-dichlorophenyl)-1',3''-dimethyl-1''-phenyl-7'',8''-dihydrodispiro[indole-3,2'-pyrrolidine-3',6''-pyrazolo[3,4-b]quinoline]-2,5''(1H,1''H)-dione chloroform solvate

Space Group:	P-1	***Cell:***	***a***	14.384(0)	***b***	14.785(0)	***c***	16.969(1)
Space Group No.:	2	***(Å,°)***	α	91.77(1)	β	96.39(1)	γ	92.15(1)

R-Factor (%): 9.64 ***Temperature(K):*** 293 ***Density(g/cm^3):*** 1.350

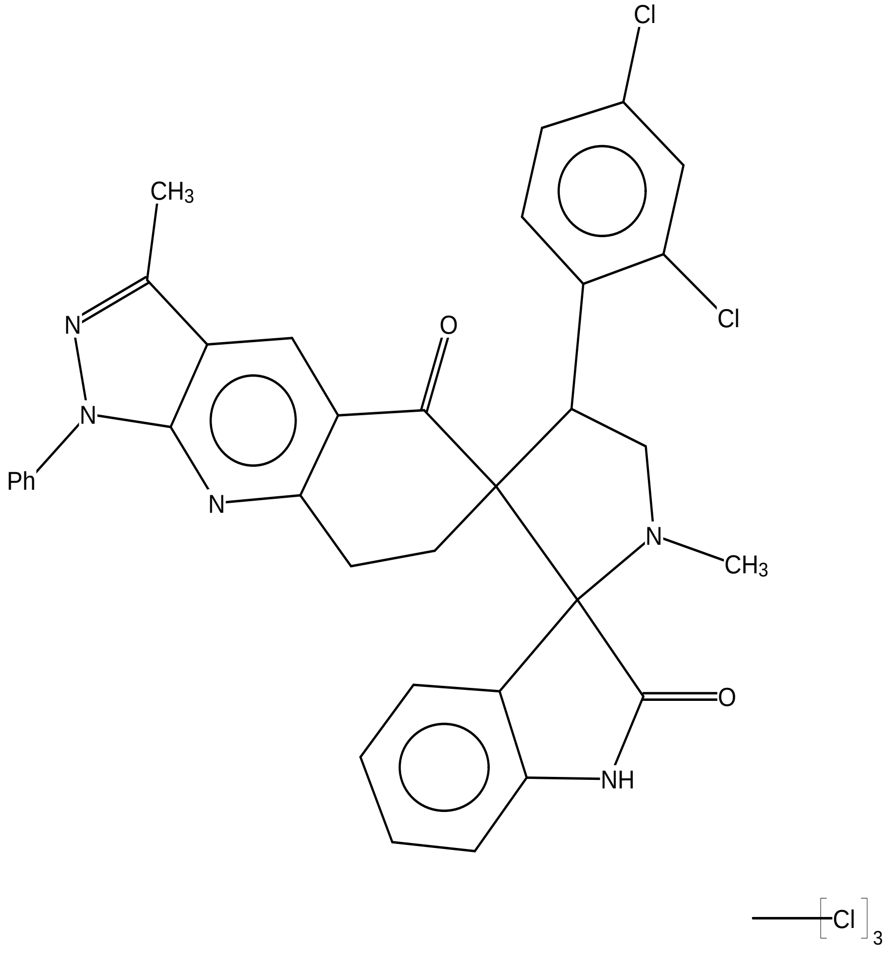

Parameters

Fragment 1
ANG1 (A) 85.568
ANG2 (A) 86.177
ANG3 (A) 51.188

Fragment 2
ANG1 (A) 85.984
ANG2 (A) 87.379
ANG3 (A) 51.545

Search: search2 (Tue Jan 01 16:11:28 2019): Hit 12

VIYQUR

Reference:	Jinlong Yan (2014) *J.Chem.Res.* ,**38,**50
Formula:	$C_{25}\ H_{21}\ Cl_1\ N_2\ O_2\ S_1$
Compound Name:	4'-(4-Chlorophenyl)-1'-methyl-6,7-dihydro-4H-dispiro[1-benzothiophene-5,3'-pyrrolidine-2',3''-indole]-2'',4(1''H)-dione

Space Group:	P-1	***Cell:***	***a***	8.217(1)	***b***	9.417(1)	***c***	14.795(1)
Space Group No.:	2	***(Å,°)***	α	71.76(0)	β	76.87(1)	γ	83.86(0)

R-Factor (%): 3.35 ***Temperature(K):*** 113 ***Density(g/cm^3):*** 1.409

Parameters

Fragment 1

**ANG1 (A)**	86.855
**ANG2 (A)**	82.781
**ANG3 (A)**	44.613

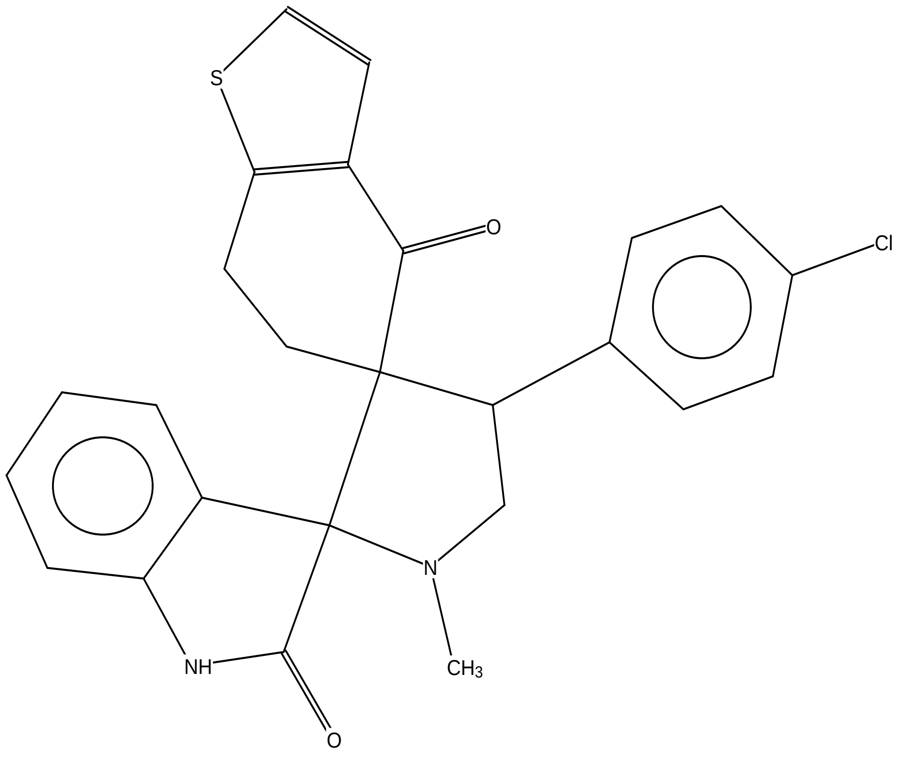

Search: search2 (Tue Jan 01 16:11:28 2019): Hit 13

YANGEC

Reference:	K.Chandralekha, D.Gavaskar, A.R.Sureshbabu, S.Lakshmi (2017) *IUCrData* ,**2,**x170639
Formula:	$C_{32} H_{30} N_2 O_4$
Compound Name:	5'-benzylidene-1''-methyl-4''-phenyl-4'H-trispiro[1,3-dioxolane-2,1'-cyclohexane-3',3''-pyrrolidine-2'',3'''-indole]-2''',4'(1'''H)-dione

Space Group:	P21/n	***Cell:***	***a***	13.960(0)	***b***	11.117(0)	***c***	16.731(0)
Space Group No.:	14	***(Å,°)***	α	90.00	β	96.02(0)	γ	90.00

R-Factor (%): 4.45 ***Temperature(K):*** 296 ***Density(g/cm^3):*** 1.303

Parameters

Fragment 1

**ANG1 (A)**	85.002
**ANG2 (A)**	87.448
**ANG3 (A)**	47.186

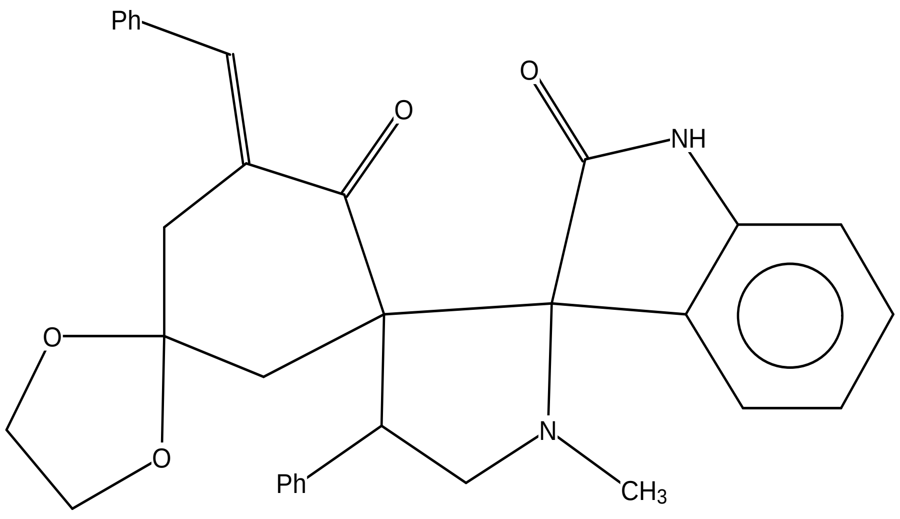

Search: search2 (Tue Jan 01 16:11:28 2019): Hit 14

VESVUN

Reference: K.Murali, H.A.Sparkes, K.J.R.Prasad (2018)
Eur.J.Med.Chem. ,**143,**292

Formula: $C_{34} H_{27} N_3 O_2$

Compound Name: 4',5'-diphenyl-4,9-dihydrodispiro[carbazole-2,3'-pyrrolidine-2',3''-indole]-1,2''(1''H,3H)-dione

Space Group:	P21/c	***Cell:***	***a*** 11.266(0)	***b*** 14.131(0)	***c*** 16.178(0)		
Space Group No.:	14	***(Å,°)***	α 90.00	β 97.00(0)	γ 90.00		

R-Factor (%): 4.30 ***Temperature(K):*** 100 ***Density(g/cm^3):*** 1.324

Parameters

Fragment 1

**ANG1 (A)**	86.821
**ANG2 (A)**	83.794
**ANG3 (A)**	42.898

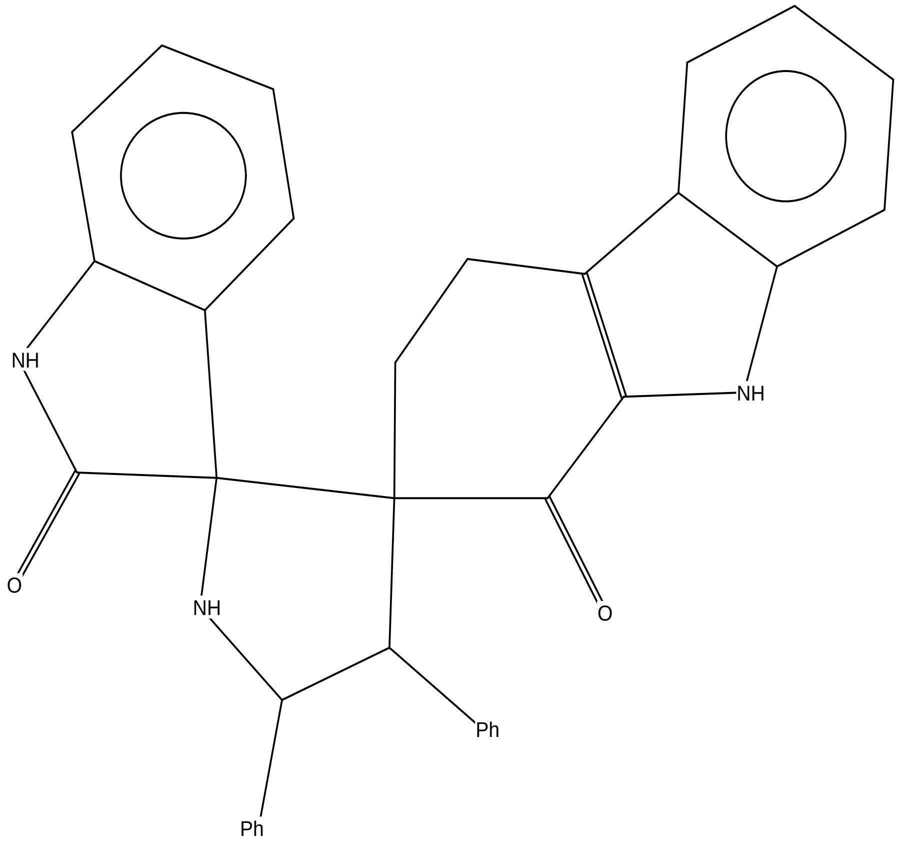

Search: search2 (Tue Jan 01 16:11:28 2019): Hit 15

JEWVOZ

Reference: Hongwen Tao, Yinan Yuan, Jian Chen, Xianyong Yu, Pinggui Yi (2018) *J.Chem.Res.* ,**42,**15

Formula: $C_{44} H_{60} N_2 O_2$

Compound Name: 1',10,13-trimethyl-17-(6-methylheptan-2-yl)-4'-phenyl-4,5,6,7,8,9,10,11,12,13,14,15,16,17-tetradecahydrodispiro[cyclopenta[a]phenanthrene-2,3'-pyrrolidine-2',3''-indole]-2'',3(1H,1''H)-dione unknown solvate

Space Group:	C2	***Cell:***	***a***	23.100(1)	***b***	9.873(0)	***c***	17.706(1)
Space Group No.:	5	***(Å,°)***	α	90.00	β	103.23(0)	γ	90.00

R-Factor (%): 4.22 ***Temperature(K):*** 293 ***Density(g/cm³):*** 1.096

Parameters

Fragment 1

**ANG1 (A)**	81.446
**ANG2 (A)**	83.751
**ANG3 (A)**	38.840

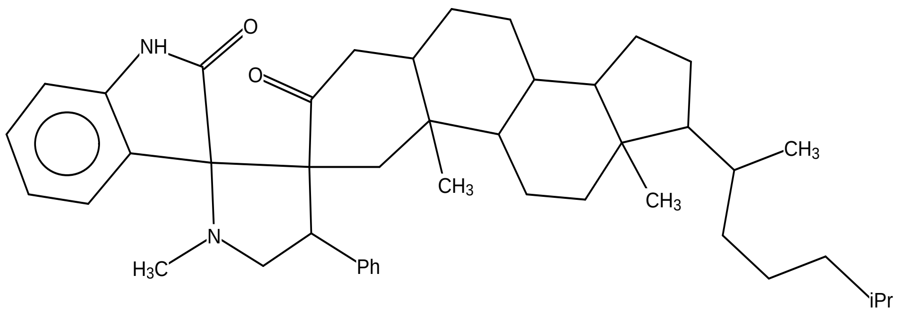

Search: search2 (Tue Jan 01 16:11:28 2019): Hit 16

KEWKAB

Reference: R.Vishnupriya, C.Selva Meenatchi, J.Suresh, R.V.Sumesh, R.R.Kumar, P.L.N.Lakshman (2018) *Acta Crystallogr.,Sect.E:Cryst.Commun.* ,**74,**660

Formula: $C_{32}\ H_{26}\ Cl_{1}\ N_{3}\ O_{2}$

Compound Name: 4'-(2-chlorophenyl)-1'-methyl-2''-phenyl-7'',8''-dihydro-5''H-dispiro[indole-3,2'-pyrrolidine-3',6''-quinoline]-2,5''(1H)-dione

Space Group:	P-1	***Cell:***	***a***	6.772(0)	***b***	11.502(0)	***c***	16.631(1)
Space Group No.:	2	***(Å,°)***	α	80.22(0)	β	84.62(0)	γ	81.08(0)

R-Factor (%): 4.73 ***Temperature(K):*** 293 ***Density(g/cm³):*** 1.373

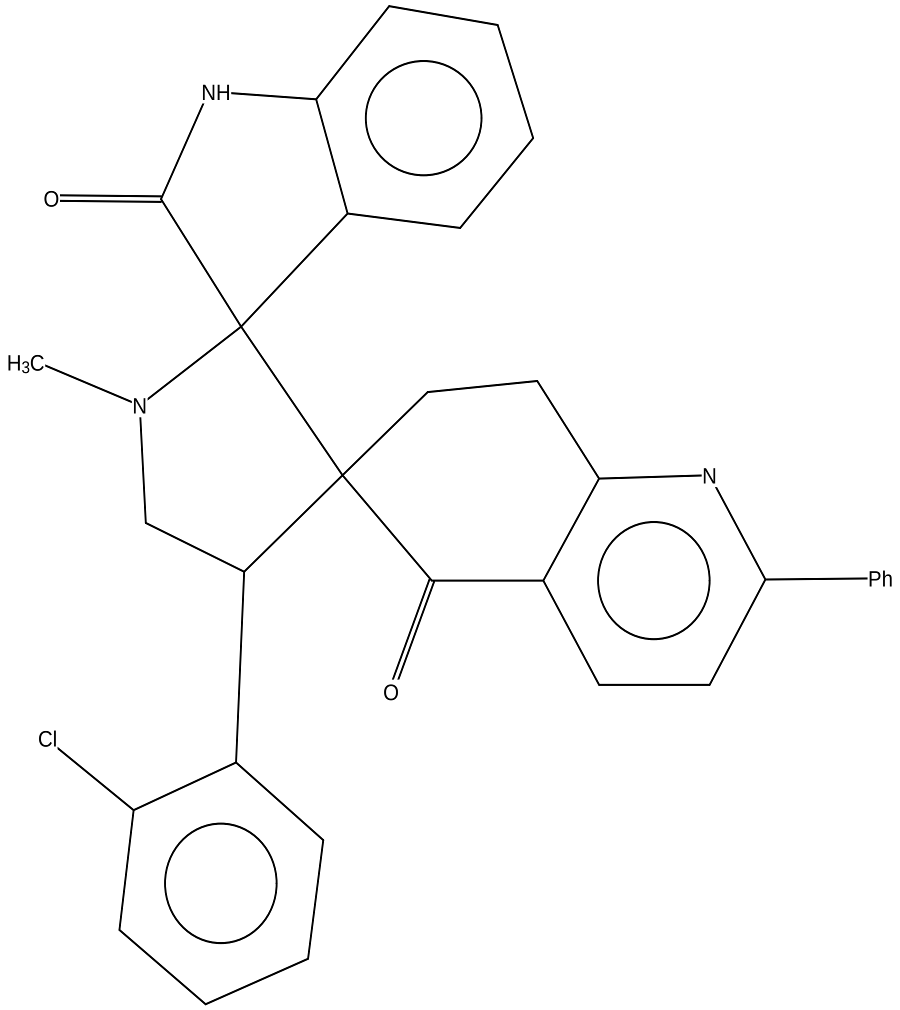

Parameters

Fragment 1

**ANG1 (A)**	86.549
**ANG2 (A)**	84.839
**ANG3 (A)**	48.226
